# A Decision Support Model for Hotel Recommendation Based on the Online Consumer Reviews Using Logarithmic Spherical Hesitant Fuzzy Information

**DOI:** 10.3390/e23040432

**Published:** 2021-04-06

**Authors:** Aziz Khan, Shougi S. Abosuliman, Saleem Abdullah, Muhammad Ayaz

**Affiliations:** 1 Department of Mathematics, Abdul Wali Khan University Mardan, Mardan 23200, Pakistan; drazizkhan9@gmail.com (A.K.); mayazmath@awkum.edu.pk (M.A.); 2 Department of Transportation and Port Management, Faculty of Maritime Studies, King Abdulaziz University, Jeddah 21588, Saudi Arabia; sabusulaiman@kau.edu.sa

**Keywords:** spherical hesitant fuzzy sets, logarithmic aggregation operators, entropy measure, decision making

## Abstract

Spherical hesitant fuzzy sets have recently become more popular in various fields. It was proposed as a generalization of picture hesitant fuzzy sets and Pythagorean hesitant fuzzy sets in order to deal with uncertainty and fuzziness information. Technique of Aggregation is one of the beneficial tools to aggregate the information. It has many crucial application areas such as decision-making, data mining, medical diagnosis, and pattern recognition. Keeping in view the importance of logarithmic function and aggregation operators, we proposed a novel algorithm to tackle the multi-attribute decision-making (MADM) problems. First, novel logarithmic operational laws are developed based on the logarithmic, t-norm, and t-conorm functions. Using these operational laws, we developed a list of logarithmic spherical hesitant fuzzy weighted averaging/geometric aggregation operators to aggregate the spherical hesitant fuzzy information. Furthermore, we developed the spherical hesitant fuzzy entropy to determine the unknown attribute weight information. Finally, the design principles for the spherical hesitant fuzzy decision-making have been developed, and a practical case study of hotel recommendation based on the online consumer reviews has been taken to illustrate the validity and superiority of presented approach. Besides this, a validity test is conducted to reveal the advantages and effectiveness of developed approach. Results indicate that the proposed method is suitable and effective for the decision process to evaluate their best alternative.

## 1. Introduction

Suppose you are looking forward to visiting any city for a while and need to book a hotel. You do not know where to stay, you think about how to make a decision about where to stay. You may be able to ask your friends, go to a travel agency, or search the Internet. All of these techniques have one thing in common: people always follow the advice of others when making decisions [1]. It is generally accepted that social networking has the potential to influence consumer purchasing decisions, both positively and negatively. Thus, contact with social networking has been a concern for marketing staff for some time [2,3]. The Internet makes it easy for consumers to share their own view of the hotel they once stayed in. For passengers, consumers normally examine the hotel on the travel website to make a comparison. Online reviews are increasingly becoming the reference information that consumers can search before making decisions, thus playing a key role in consumer decision-making.

With the rapid growth of internet technology [4], like Web 2.0, hotels can be easily selected on the web site. Web 2.0 innovations have changed the way consumers search for hotels significantly. It offers consumers the convenience of accessing the goods and services of hotels at any time and in any location. Therefore, with the advent of an online booking system, travelers around the world are able to enter targeted hotels by clicking [5]. The online sequence of hotels is very critical because it can influence consumers’ booking decisions [6]. In fact, the tourism website’s online reviews play a major role in directing people’s everyday consumption [7]. In addition, each consumer relies on the hotel evaluation requirements, and when sorting the hotels, it is best to take a detailed account. That is why most of the new hotel sequences on the tourism website are constrained in terms of meeting consumers’ expectations [8]. Researchers in this area are therefore starting to concentrate on proposing a form of hotel recommendation that can help customers easily and reliably locate the most suitable hotel [9]. At present, online reviews from both theoretical and realistic viewpoints have been reviewed by several researchers. Online reviews were listed in [10] and observed that online reviews are strongly skewed toward positive scores. Ye et al. [11], Spark and Browning [1], and Simmons et al. [12] presented the role of online reviews for hotel booking in different style. The customer satisfaction of the hotel was obtained by Berezina et al. [13] through the process of text-link analysis. Some application research on hotel recommendation problems is also available [10,14]. In addition, it is suggested that online feedback of similar groups can have a substantial influence on decision-making. Moreover, granular computing [15,16,17] is commonly used in related group clustering. While researchers have proposed the concept of fuzzy sets and applied them to several fields [18,19,20], like hotel ranking [21,22], all of the details in online reviews, in particular text types, cannot be protected. Actually, Thong [23] found out that the emotions reflected in text reviews were expensive.

The multi-attribute group decision-making (MAGDM) process is a significant and emerging issue that illustrates a method for selecting the best alternative with a group of decision-makers (DMs) and situations. There are two severe goals in this technique: The first goal is to describe the environment in which the values of a few attributes can be effectively analyzed, while the second goal is to aggregate the described data. Generally, the data that describe the substances are taken in the form of deterministic or crisp in nature. However, as the structures become more difficult to manage, collecting data from records, assets, and professionals in a clear and concise manner becomes increasingly difficult. Thus, in order to express information more flexibly, the researchers employ the concept of fuzzy sets [24] and their generalizations like intuitionistic fuzzy set (IFSs) [25,26,27,28,29], Pythagorean fuzzy sets (PyFSs) [30,31], hesitant fuzzy sets (HFSs) [32], picture fuzzy sets [33,34,35,36], etc.

Spherical fuzzy sets (SFSs), proposed by Ashraf et al. [37,38,39], are a generalized structure of the all existing structures of fuzzy sets. SFSs can handle vagueness more successfully and competently in decision-making problems (DMPs). Ashraf et al. [40] established the spherical fuzzy Dombi aggregation operators (AOs) under spherical fuzzy information. Jin et al. [41] presented the linguistic spherical fuzzy AOs under SF information. Rafiq et al. [42] presented the decision-making technique based on the cosine similarity measures to tackle the uncertainty in real-life DMPs. Ashraf et al. [43] developed the spherical distance measure under SF settings. Ashraf et al. [44] proposed the spherical fuzzy set representation using t-norm and t-conorm. Zeng et al. [45] established the spherical fuzzy rough set structure and proposed the TOPSIS methodology to tackle the inaccurate and uncertain information in the form of SFSs. Jin et al. [46] presented the logarithmic function-based AOs under spherical fuzzy settings. Ashraf et al. [47] introduced the novel AOs based on symmetric sum under spherical fuzzy settings. Barukab et al. [48] established the advanced fuzzy TOPSIS tachnique under spherical fuzzy environment to tackle the uncertainty in DMPs. Ashraf et al. [49,50] presented the spherical fuzzy set based emergency decision-making methodology to tackle the uncertainty in emergency situation.

To overwhelm the hesitancy, Torra [51] recognized the notion of FSs with hesitancy. By means of hesitant fuzzy set (HFS), many writers determined problems by aggregating the operators in group decision-making: Liu and Sun [52] presented the power average AOs under HFS environment and discussed their application in DMPs. Xia and Xu [53] developed the novel aggregation information to tackle the uncertainty in DMPs. Khan et al. [54] discussed the applications of probabilistic hesitant fuzzy rough set in decision aid system. Afterwards, Khan et al. [55] recognized the idea of Pythagorean HFS (PyHFS). They presented an assessment method and recognized operators to aggregate the data. Khan et al. [56] recognized Pythagorean hesitant fuzzy weighted average and hybrid aggregation operators and their application to DMPs. Recently, Naeem et al. [57] introduced the novel concept of spherical hesitant fuzzy sets, which is the hybrid structure of hesitant fuzzy sets and spherical fuzzy sets. Spherical hesitant fuzzy sets (SHFSs) is the triplet having positive, neutral, and negative membership grades in the form of sets consider some values in $\left[0,1\right].$ As SHFS is very effective and reliable to tackle the hesitancy in real life decision-making problems. Therefore, motivated by the concept of SHFSs, in this paper, we developed the novel aggregation operators based on the logarithmic function. The main contribution of the article is listed as follows:

(1) Novel logarithmic operational laws under spherical hesitant fuzzy numbers are developed.
(2) Based on the logarithmic operational laws, a novel list of algebraic aggregation operators is introduced to aggregate the uncertain information in real word decision making problems.
(3) A decision-making algorithm is presented to deal decision-making problems.
(4) A real-life decision-making problem of hotel selection is illustrated using proposed algorithm.
(5) A validity test is given to show the effectiveness and reliability of the proposed methodology.

The rest of this paper is organized as follows. In Section 2, basic studies on generalizations of fuzzy sets are briefly reviewed. In Section 3, the basic knowledge about logarithmic operational laws are given. In Section 4, we propose list of novel logarithmic spherical hesitant fuzzy aggregation operators and their related properties. In Section 5, we provide a decision-making algorithm to tackle the real life decision-making problems. In Section 6, a case study about hotel selection is illustrated to show the applicability of the proposed methodology. In Section 7, a comparison study is presented, also a validity test is presented in Section 8, to show the effectiveness and reliability of the developed approach. Finally, conclusion is drawn in Section 9.

## 2. Preliminaries

In this section, studies on generalizations of fuzzy sets are briefly reviewed.

**Definition** **1.** *[24] Suppose the ground set N≠ϕ. A fuzzy set (FS) C is described as below,*

(1) $$C=\{\langle q,E_{C}\left(q\right)\rangle |q\in N\},$$

*where $E_{C}\left(q\right)\in \left[0,1\right]$ indicate the membership grade of q in C.*

**Definition** **2.** *[51] Suppose the ground set N≠ϕ. A hesitant FS (HFS) C is described as below,*

(2) $$C=\{\langle q,h_{C}\left(q\right)\rangle |q\in N\},$$

*where $h_{C}\left(q\right)$ be any set having the some values in $\left[0,1\right].$*

**Definition** **3.** *[25] Suppose the ground set N≠ϕ. An intuitionistic FS C is described as below,*

(3) $$C=\{\langle q,E_{C}\left(q\right),F_{C}\left(q\right)\rangle |q\in N\},$$

*where $E_{C}:q\to \left[0,1\right]$ be positive and $F_{C}:q\to \left[0,1\right]$ be negative membership grades with the constraint $E_{C}\left(q\right)+F_{C}\left(q\right)\leq 1,$∀q∈N.*

**Definition** **4.** *[30] Suppose the ground set N≠ϕ. A Pythagorean FS C is described as below,*

(4) $$C=\{\langle q,M_{C}\left(q\right),K_{C}\left(q\right)\rangle |q\in N\},$$

*where $M_{C}:g\to \left[0,1\right]$ be positive and $K_{C}:q\to \left[0,1\right]$ be negative membership grades with the constraint ${\left(M_{C}\left(q\right)\right)}^{2}+{\left(K_{C}\left(q\right)\right)}^{2}\leq 1,$∀q∈N.*

**Definition** **5.** *[33] Suppose the ground set N≠ϕ. A picture FS C is described as below,*

(5) $$C=\{\langle q,E_{C}\left(q\right),F_{C}\left(q\right),G_{C}\left(q\right)\rangle |q\in N\},$$

*where $E_{C}:q\to \left[0,1\right]$ be positive, $F_{C}:q\to \left[0,1\right]$ be neutral, and $G_{C}:q\to \left[0,1\right]$ be negative membership grades with the constraint $\left(E_{C}\left(q\right)\right)+\left(F_{C}\left(q\right)\right)+\left(G_{C}\left(q\right)\right)\leq 1,$∀ q∈N.*

**Definition** **6.** *[37,38,39] Suppose the ground set N≠ϕ. A spherical FS C is described as below,*

(6) $$C=\{\langle q,E_{C}\left(q\right),F_{C}\left(q\right),G_{C}\left(q\right)\rangle |q\in N\},$$

*where $E_{C}:q\to \left[0,1\right]$ be positive, $F_{C}:q\to \left[0,1\right]$ be neutral, and $G_{C}:q\to \left[0,1\right]$ be negative membership grades with the constraint ${\left(E_{C}\left(q\right)\right)}^{2}+{\left(F_{C}\left(q\right)\right)}^{2}+{\left(G_{C}\left(q\right)\right)}^{2}\leq 1,$∀ q∈N.*

**Definition** **7.** *[57] Suppose the ground set N≠ϕ. A spherical hesitant FS (SHFS) C is described as below,*

(7) $$C=\{\langle q,E_{C}\left(q\right),F_{C}\left(q\right),G_{C}\left(q\right)\rangle |q\in N\},$$

*where*

$$E_{C}\left(q\right)=\left\{u|u\in \left[0,1\right]\right\},\;F_{C}\left(q\right)=\left\{v|v\in \left[0,1\right]\right\}\;and\;G_{C}\left(q\right)=\left\{w|w\in \left[0,1\right]\right\},$$

*are the three sets of some values in [0,1], denoted the positive, neutral, and negative membership grades with the constraint $0\leq {\left(u^{+}\right)}^{2}+{\left(v^{+}\right)}^{2}+{\left(w^{+}\right)}^{2}\leq 1,$∀q∈N, such that*

$$u^{+}=\underset{u\in E_{C}\left(q\right)}{⋃}\max\left\{u\right\},\;v^{+}=\underset{v\in F_{C}\left(q\right)}{⋃}\max\left\{v\right\},\;and\;w^{+}=\underset{\;\;w\in G_{C}\left(q\right)}{⋃}\max\left\{w\right\}.$$

For easiness, we signified $SHFS\left(C\right)$ be the list of spherical hesitant FSs and the triplet $\left(E_{C},F_{C},G_{C}\right)$ is called spherical hesitant fuzzy number (SHFN).

**Definition** **8.** *[57] Suppose $C_{q}=\left\{E_{q},F_{q},G_{q}\right\}$$\in SHFN\left(N\right)$$\left(q\in N\right).$ The basic operational laws are described as below,*

*(1) ${\left(C_{1}\right)}^{c}=\underset{\left(u_{1},v_{1},w_{1}\right)\in \left(E_{1},F_{1},G\lambda_{1}\right)}{⋃}\left\{E_{1},F_{1},G_{1}\right\};$*

*(2) $C_{1}\cup C_{2}=\underset{\left(u_{q},v_{q},w_{q}\right)\in \left(M_{q},F_{q},G_{q}\right)}{⋃}\left\{\max\left(E_{q}\right),\min\left(F_{q}\right),\min\left(G_{q}\right)\right\};$*

*(3) $C_{1}\cap C_{2}=\underset{\left(u_{q},v_{q},w_{q}\right)\in \left(M_{q},F_{q},G_{q}\right)}{⋃}\left\{\min\left(E_{q}\right),\min\left(F_{q}\right),\max\left(G_{q}\right)\right\};$*

**Definition** **9.** *[57] Let C={E,F,G}, $C_{1}=\left\{E_{1},F_{1},G_{1}\right\}$, and $C_{2}=\left\{E_{2},F_{2},G_{2}\right\}$ be the three SHFEs, β>1. Then, the operational laws for SHFNs are described as*

*(1) $C_{1}⊕C_{2}=\;\;\underset{\begin{array}{c} u\in E_{1},v_{1}\in F_{1},w_{1}\in G_{1}\\ u_{2}\in E_{2},v_{2}\in F,w_{2}\in G_{1} \end{array}}{⋃}\left\{\sqrt{u_{1}^{2}+u_{2}^{2}-u_{1}^{2}u_{2}^{2}},v_{1}v_{2},w_{1}w_{2}\right\};$*

*(2) $C_{1}⊗C_{2}=\;\underset{\begin{array}{c} u\in E_{1},v_{1}\in F_{1},w_{1}\in G_{1}\\ u_{2}\in E_{2},v_{2}\in F,w_{2}\in G_{1} \end{array}}{⋃}\;\left\{\{u_{1}u_{2},v_{1}v_{2},\sqrt{w_{1}^{2}+w_{2}^{2}-w_{1}^{2}w_{2}^{2}}\right\};$*

*(3) $\beta C=\underset{u\in E,v\in F,w\in G}{⋃}\left\{\sqrt{1-{\left(1-u^{2}\right)}^{\beta }},{\left(v\right)}^{\beta },{\left(w\right)}^{\beta }\right\};$*

*(4) $C^{\beta }=\underset{u\in E,v\in F,w\in G}{⋃}\left\{{\left(u\right)}^{\beta },{\left(v\right)}^{\beta },\sqrt{1-{\left(1-w^{2}\right)}^{\beta }}\right\}.$*

**Definition** **10.** *Let C={E,F,G} be a SHFN, then the score function S of C is defined as*

(8) $$S\left(k\right)=\frac{1}{l\left(E_{C}\right)}\sum E_{C}-\frac{1}{l\left(F_{C}\right)}\sum F_{C}-\frac{1}{l\left(G_{C}\right)}\sum G_{C}\;.\;$$

*where l represented the number of elements in membership grades.*

**Definition** **11.** *Let C={E,F,G} be a SHFN, then the accuracy function H is defined as*

$$H\left(C\right)=E_{C}+F_{C}+G_{C}.$$

On the basis of score and accuracy functions, a comparison system is specified as

**Definition** **12.** *Let $C_{1}$ and $C_{2}$ be two SHFNs, $S(C_{i})$ is the score function and $H(C_{i})$ is the accuracy function of $C_{i}\left(i=1,2\right)$, then*

*(1) If $S\left(C_{1}\right)>S\left(C_{2}\right)$, then $C_{1}>C_{2}$;*

*(2) If $S\left(C_{1}\right)=S\left(C_{2}\right)$, then*

*(a) If $H\left(C_{1}\right)>H\left(C_{2}\right)$, then $C_{1}>C_{2}$;*

*(b) If $H\left(C_{1}\right)=H\left(C_{2}\right)$, then $C_{1}=C_{2}$;*

*(c) If $H\left(C_{1}\right)<H\left(C_{2}\right)$, then $C_{1}<C_{2}.$*

**Definition** **13.** *[38,39] Suppose $C_{q}=\left\{E_{q},F_{q},G_{q}\right\}$$\in SFN\left(N\right)$$\left(q\in N\right).$ Then, the weighted averaging operator for SFNs is described as*

(9) $$\begin{array}{ccc} SFWA\left(C_{1},C_{2},\ldots C_{m}\right) & = & \beta_{1}C_{1}⊕\beta_{2}C_{n}⊕\ldots ⊕\beta_{m}C_{m}\\ & = & \sum_{q=1}^{m}\beta_{q}C_{q} \end{array}$$

*where $\beta ={\left(\beta_{1},\beta_{2},\ldots \beta_{m}\right)}^{T}$ is weight information of $\left(C_{1},C_{2},\ldots C_{m}\right)$ such that $\beta_{q}\geq 0$; $\sum_{q=1}^{m}\beta_{q}=1.$*

**Definition** **14.** *[38,39] Suppose $C_{q}=\left\{E_{q},F_{q},G_{q}\right\}$$\in SFN\left(N\right)$$\left(q\in N\right).$ Then, ordered weighted averaging operator for SFNs is described as*

(10) $$\begin{array}{ccc} SFOWA\left(C_{1},C_{2},\ldots C_{m}\right) & = & \beta_{1}C_{\ell \left(1\right)}⊕\beta_{2}C_{\ell \left(2\right)}⊕\ldots ⊕\beta_{m}C_{\ell \left(m\right)}\\ & = & \sum_{q=1}^{m}\beta_{q}C_{\ell \left(q\right)} \end{array}$$

*where $\ell \left(q\right)$ denote the order according to $\left(\ell \left(1\right),\ell \left(2\right),\ell \left(3\right),\ldots ,\ell \left(m\right)\right)$ and $\beta ={\left(\beta_{1},\beta_{2},\ldots \beta_{m}\right)}^{T}$ is weight information of $\left(C_{1},C_{2},\ldots C_{m}\right)$ such that $\beta_{q}\geq 0$; $\sum_{q=1}^{m}\beta_{q}=1.$*

**Definition** **15.** *[38,39] Suppose $C_{q}=\left\{E_{q},F_{q},G_{q}\right\}$$\in SFN\left(N\right)$$\left(q\in N\right).$ Then, weighted geometric operator for SFNs is described as*

(11) $$\begin{array}{ccc} SHFWG\left(C_{1},C_{2},\ldots C_{m}\right) & = & {\left(C_{1}\right)}^{\beta_{1}}⊗{\left(C_{2}\right)}^{\beta_{2}}⊗\ldots ⊗{\left(C_{m}\right)}^{\beta_{m}}\\ & = & \prod_{q=1}^{m}{\left(C_{q}\right)}^{\beta_{q}} \end{array}$$

*where ${\left(\beta_{1},\beta_{2},\ldots \beta_{m}\right)}^{T}$ is weight information of $\left(C_{1},C_{2},\ldots C_{m}\right)$ such that $\beta_{q}\geq 0$; $\sum_{q=1}^{m}\beta_{q}=1.$*

**Definition** **16.** *[38,39] Suppose $C_{q}=\left\{E_{q},F_{q},G_{q}\right\}$$\in SFN\left(N\right)$$\left(q\in N\right).$ Then, ordered weighted geometric operator for SFNs is described as*

(12) $$\begin{array}{ccc} SHFOWG\left(C_{1},C_{2},\ldots C_{m}\right) & = & {\left(C_{\ell \left(1\right)}\right)}^{\beta_{1}}⊗{\left(C_{\ell \left(2\right)}\right)}^{\beta_{2}}⊗\ldots ⊗{\left(C_{\ell \left(m\right)}\right)}^{\beta_{m}}\\ & = & \prod_{q=1}^{m}{\left(C_{\ell \left(q\right)}\right)}^{\beta_{q}} \end{array}$$

*where $\ell \left(q\right)$ is represented the order according to $\left(\ell \left(1\right),\ell \left(2\right),\ell \left(3\right),\ldots ,\ell \left(m\right)\right)$ and ${\left(\beta_{1},\beta_{2},\ldots \beta_{m}\right)}^{T}$ is weight information of $\left(C_{1},C_{2},\ldots C_{m}\right)$ such that $\beta_{q}\geq 0$; $\sum_{q=1}^{m}\beta_{q}=1.$*

## 3. Operational Laws for Logarithmic Spherical Hesitant Fuzzy Sets

**Definition** **17.** *Suppose $C_{q}=\left\{E_{q},F_{q},G_{q}\right\}$$\in SHFN\left(N\right)$. A Logarithmic spherical hesitant FS (LSHFS) is described as below,*

(13) $$Log_{i}C_{q}=\left\{\sqrt{1-{\left(Log_{{}_{i}}E_{C}\left(q\right)\right)}^{2}},Log_{{}_{i}}\left(\sqrt{1-F_{C}^{2}\left(q\right)}\right),Log_{{}_{i}}\left(\sqrt{1-G_{C}^{2}\left(q\right)}\right)|q\in N\right\}$$

*where $Log_{{}_{i}}E_{C}\left(q\right)=\left\{u|u\in \left[0,1\right]\right\},$$Log_{{}_{i}}\left(1-F_{C}\left(q\right)\right)=\left\{v|v\in \left[0,1\right]\right\}$ and $Log_{{}_{i}}\left(1-G_{C}\left(q\right)\right)=\left\{w|w\in \left[0,1\right]\right\}$ are the three sets of some values in [0,1], denoted the positive, neutral, and negative membership grades with the constraint $0\leq {\left(u^{+}\right)}^{2}+{\left(v^{+}\right)}^{2}+{\left(w^{+}\right)}^{2}\leq 1,$ for all q∈N, such that*

$$\begin{array}{cc} u^{+}=\underset{u\in \left(1-Log_{{}_{i}}E_{C}\left(q\right)\right)}{⋃}\max\left\{u\right\}, & v^{+}={⋃}_{v\in Log_{{}_{i}}\left(1-F_{C}\left(q\right)\right)}\max\left\{v\right\},\; \end{array}$$

*and*

$$w^{+}=\underset{\;\;w\in Log_{{}_{i}}\left(1-G_{C}\left(q\right)\right)}{⋃}\max\left\{w\right\}.$$

**Definition** **18.** *Suppose $C_{q}=\left\{E_{q},F_{q},G_{q}\right\}$$\in SHFN\left(N\right)$. If*

$$Log_{i}C_{q}=\left\{\begin{array}{cc} \left(\begin{array}{c} \sqrt{1-{\left(Log_{{}_{i}}E_{C}\left(q\right)\right)}^{2}},\\ Log_{{}_{i}}\left(\sqrt{1-F_{C}^{2}\left(q\right)}\right),\\ Log_{{}_{i}}\left(\sqrt{1-G_{C}^{2}\left(q\right)}\right) \end{array}\right) & 0<i\leq \min\left\{E_{C},\sqrt{1-F_{C}^{2}},\sqrt{1-G_{C}^{2}}\right\}<1\\ \left(\begin{array}{c} \sqrt{1-{\left(Log_{\frac{1}{i}}E_{C}\left(q\right)\right)}^{2}},\\ Log_{\frac{1}{i}}\left(\sqrt{1-F_{C}^{2}\left(q\right)}\right),\\ Log_{\frac{1}{i}}\left(\sqrt{1-G_{C}^{2}\left(q\right)}\right) \end{array}\right) & 0<\frac{1}{i}\leq \min\left\{E_{C},\sqrt{1-F_{C}^{2}},\sqrt{1-G_{C}^{2}}\right\}<1 \end{array}\right.$$

*then $Log_{i}C_{q}$ is called logarithmic operator for spherical hesitant fuzzy set. Here, we take $Log_{i}0=0,$i>0 and i≠1.*

**Theorem** **1.** *Let $C_{q}=\left\{E_{q},F_{q},G_{q}\right\}\in SHFN\left(N\right)$, then $Log_{i}C_{q}$ is also a SHFN.*

**Proof.**  As we know that for $C_{q}=\left\{E_{q},F_{q},G_{q}\right\}$ in N we have $E_{q}:N\to \left[0,1\right],F_{q}:N\to \left[0,1\right]$ and $G_{q}:N\to \left[0,1\right]$ denote the positive, negative and neutral membership degrees. Furthermore, the following constraint holds:

$$0\leq E_{C}^{2}\left(q\right)+F_{C}^{2}\left(q\right)+G_{C}^{2}\left(q\right)\leq 1.$$

The following two cases will also happen:

**Case-1** When

$$0<i\leq \min\left\{E_{C},\sqrt{1-F_{C}^{2}},\sqrt{1-G_{C}^{2}}\right\}<1.i\neq 1,$$

since $Log_{i}C_{q}$ is a decreasing function w.r.t i. Therefore,

$$0\leq Log_{i}E_{C},Log_{i}\left(\sqrt{1-F_{C}^{2}}\right),Log_{i}\left(\sqrt{1-G_{C}^{2}}\right)<1,$$

and

$$0\leq \sqrt{1-{\left(Log_{{}_{i}}E_{C}\right)}^{2}}\leq 1,0\leq Log_{{}_{i}}\left(\sqrt{1-F_{C}^{2}}\right)\leq 1,0\leq Log_{{}_{i}}\left(\sqrt{1-G_{C}^{2}}\right)\leq 1,$$

and

$$0\leq \sqrt{1-{\left(Log_{{}_{i}}E_{C}\right)}^{2}}+Log_{i}\left(\sqrt{1-F_{C}^{2}}\right)+Log_{i}\left(\sqrt{1-G_{C}^{2}}\right)\leq 1.$$

Hence, $Log_{i}C_{q}$ is a SHFN.

**Case-2** When $i>1,0<\frac{1}{i}<1$ and

$$\frac{1}{i}\leq \min\left\{E_{C},\sqrt{1-F_{C}^{2}},\sqrt{1-G_{C}^{2}}\right\}<1.$$

By the same approach as in case-1, we can prove that $Log_{i}C_{q}$ is a SHFN.  □

**Definition** **19.** *Let $Log_{i}C_{q}=\left\{\sqrt{1-{\left(Log_{{}_{i}}E_{q}\right)}^{2}},Log_{{}_{i}}\left(\sqrt{1-F_{q}^{2}}\right),Log_{{}_{i}}\left(\sqrt{1-G_{q}^{2}}\right)\right\}$$\in SHFN\left(N\right)$, β>1. Then, the operations for LSHFNs are described as*

*(1) $Log_{{}_{i}}C_{1}⊕Log_{{}_{i}}C_{2}=\;\;\underset{\begin{array}{c} u_{q}\in \left(\sqrt{1-{\left(Log_{{}_{i}}E_{q}\right)}^{2}}\right),\\ v_{q}\in Log_{{}_{i}}\left(\sqrt{1-F_{q}^{2}}\right),\;w_{q}\in Log_{{}_{i}}\left(\sqrt{1-G_{q}^{2}}\right) \end{array}}{⋃}\left\{\begin{array}{c} \sqrt{1-{\left(Log_{{}_{i}}u_{1}\right)}^{2}{\left(Log_{{}_{i}}u_{2}\right)}^{2}},\\ Log_{{}_{i}}\sqrt{\left(1-v_{1}^{2}\right)}.Log_{{}_{i}}\sqrt{\left(1-v_{2}^{2}\right)},\\ Log_{{}_{i}}\sqrt{\left(1-w_{1}^{2}\right)}.Log_{{}_{i}}\sqrt{\left(1-w_{2}^{2}\right)} \end{array}\right\};$*

*(2) $Log_{{}_{i}}C_{1}⊗Log_{{}_{i}}C_{2}$*

$=\;\underset{\begin{array}{c} u_{q}\in \left(\sqrt{1-{\left(Log_{{}_{i}}E_{q}\right)}^{2}}\right),\;v_{q}\in Log_{{}_{i}}\left(\sqrt{1-F_{q}^{2}}\right),\\ w_{q}\in Log_{{}_{i}}\left(\sqrt{1-G_{1}^{2}}\right) \end{array}}{⋃}\;\left\{\begin{array}{c} \sqrt{1-{\left(Log_{{}_{i}}u_{1}\right)}^{2}}\sqrt{1-{\left(Log_{{}_{i}}u_{2}\right)}^{2}},\\ \sqrt{1-{\left(1-Log_{{}_{i}}\left(\sqrt{1-v_{1}^{2}}\right)\right)}^{2}.{\left(1-Log_{{}_{i}}\left(\sqrt{1-v_{2}^{2}}\right)\right)}^{2}},\\ \sqrt{1-{\left(1-Log_{{}_{i}}\left(\sqrt{1-w_{1}^{2}}\right)\right)}^{2}.{\left(1-Log_{{}_{i}}\left(\sqrt{1-w_{2}^{2}}\right)\right)}^{2}} \end{array}\right\};$

*(3) $\beta Log_{{}_{i}}C_{1}=\underset{\begin{array}{c} u_{1}\in \left(\sqrt{1-{\left(Log_{{}_{i}}E_{1}\right)}^{2}}\right),\;v_{1}\in Log_{{}_{i}}\left(\sqrt{1-F^{2}}\right),\\ w_{1}\in Log_{{}_{i}}\left(\sqrt{1-G^{2}}\right) \end{array}}{⋃}\left\{\begin{array}{c} \sqrt{1-{\left(Log_{{}_{i}}u_{1}\right)}^{2\beta }},Log_{{}_{i}}{\left(\sqrt{1-v_{1}^{2}}\right)}^{\beta },\\ Log_{{}_{i}}{\left(\sqrt{1-w_{1}^{2}}\right)}^{\beta } \end{array}\right\};$*

*(4) ${\left(Log_{{}_{i}}C_{1}\right)}^{\beta }=\underset{\begin{array}{c} u_{1}\in \left(\sqrt{1-{\left(Log_{{}_{i}}E_{1}\right)}^{2}}\right),\;v_{1}\in Log_{{}_{i}}\left(\sqrt{1-F^{2}}\right),\\ w_{1}\in Log_{{}_{i}}\left(\sqrt{1-G^{2}}\right) \end{array}}{⋃}\left\{\begin{array}{c} {\left(\sqrt{1-{\left(Log_{{}_{i}}u_{1}\right)}^{2}}\right)}^{\beta },\\ \sqrt{1-{\left(1-{\left(Log_{{}_{i}}\sqrt{1-v_{1}^{2}}\right)}^{2}\right)}^{\beta }},\\ \sqrt{1-{\left(1-{\left(Log_{{}_{i}}\sqrt{1-w_{1}^{2}}\right)}^{2}\right)}^{\beta }} \end{array}\right\}.$*

**Theorem** **2.** *Suppose $C_{q}=\left\{E_{q},F_{q},G_{q}\right\}\in SHFN\left(N\right)$ with $0<i\leq \min\left\{\begin{array}{c} E_{C},\\ \sqrt{1-F_{C}^{2}},\\ \sqrt{1-G_{C}^{2}}<1 \end{array}\right\},$i≠1, then*

*(1) $i^{Log_{{}_{i}}C}=C;$*

*(2) $Log_{{}_{i}}i^{C}=C.$*

**Proof.**  $\left(1\right)$ By using Definition 19, we have

$$i^{Log_{{}_{i}}C}=\underset{\begin{array}{c} u\in \left(\sqrt{1-{\left(Log_{{}_{i}}E\right)}^{2}}\right),v\in Log_{{}_{i}}\left(\sqrt{1-F^{2}}\right),\\ w\in Log_{{}_{i}}\left(\sqrt{1-G^{2}}\right) \end{array}}{⋃}\left(\begin{array}{c} i^{\sqrt{1-{\left(\sqrt{1-{\left(Log_{{}_{i}}u\right)}^{2}}\right)}^{2}}},\\ \sqrt{1-i^{2Log_{{}_{i}}\sqrt{1-v^{2}}}},\\ \sqrt{1-i^{2Log_{{}_{i}}\sqrt{1-w^{2}}}} \end{array}\right);$$

$$\begin{array}{ccc} & = & \underset{\begin{array}{c} u\in \left(\sqrt{1-{\left(Log_{{}_{i}}E\right)}^{2}}\right),v\in Log_{{}_{i}}\left(\sqrt{1-F^{2}}\right),\\ w\in Log_{{}_{i}}\left(\sqrt{1-G^{2}}\right) \end{array}}{⋃}\left(\begin{array}{c} i^{\sqrt{1-\left(1-{\left(Log_{{}_{i}}u\right)}^{2}\right)}},\\ \sqrt{1-\left(1-v^{2}\right)},\\ \sqrt{1-\left(1-w^{2}\right)} \end{array}\right);\\ & = & \underset{u\in \left(\sqrt{1-{\left(Log_{{}_{i}}E\right)}^{2}}\right),v\in Log_{{}_{i}}\left(\sqrt{1-F^{2}}\right),w\in Log_{{}_{i}}\left(\sqrt{1-G^{2}}\right)}{⋃}\left(i^{Log_{{}_{i}}u},v,w\right);\\ & = & \underset{u\in \left(\sqrt{1-{\left(Log_{{}_{i}}E\right)}^{2}}\right),v\in Log_{{}_{i}}\left(\sqrt{1-F^{2}}\right),w\in Log_{{}_{i}}\left(\sqrt{1-G^{2}}\right)}{⋃}\left(u,v,w\right)=C. \end{array}$$

(2) By using Definition 19, we have

$$Log_{{}_{i}}i^{C}=\underset{\begin{array}{c} u\in \left(\sqrt{1-{\left(Log_{{}_{i}}E\right)}^{2}}\right),v\in Log_{{}_{i}}\left(\sqrt{1-F^{2}}\right),\\ w\in Log_{{}_{i}}\left(\sqrt{1-G^{2}}\right) \end{array}}{⋃}Log_{{}_{i}}\left\{\left(\begin{array}{c} i^{\sqrt{1-u^{2}}},\\ \sqrt{1-i^{2v}},\\ \sqrt{1-i^{2w}} \end{array}\right)\right\};$$

$$\begin{array}{ccc} & = & \underset{\begin{array}{c} u\in \left(\sqrt{1-{\left(Log_{{}_{i}}E\right)}^{2}}\right),v\in Log_{{}_{i}}\left(\sqrt{1-F^{2}}\right),\\ w\in Log_{{}_{i}}\left(\sqrt{1-G^{2}}\right) \end{array}}{⋃}\left\{\begin{array}{c} \sqrt{1-{\left(Log_{{}_{i}}i^{\sqrt{1-u^{2}}}\right)}^{2}},\\ Log_{{}_{i}}\sqrt{1-{\left(\sqrt{1-i^{2v}}\right)}^{2}},\\ Log_{{}_{i}}\sqrt{1-{\left(\sqrt{1-i^{2v}}\right)}^{2}} \end{array}\right\};\\ & = & \underset{\begin{array}{c} u\in \left(\sqrt{1-{\left(Log_{{}_{i}}E\right)}^{2}}\right),v\in Log_{{}_{i}}\left(\sqrt{1-F^{2}}\right),\\ w\in Log_{{}_{i}}\left(\sqrt{1-G^{2}}\right) \end{array}}{⋃}\left\{\begin{array}{c} \sqrt{1-\left(1-u^{2}\right)},Log_{{}_{i}}\sqrt{1-\left(1-i^{2v}\right)},\\ Log_{{}_{i}}\sqrt{1-\left(1-i^{2v}\right)} \end{array}\right\};\\ \; & = & \left(u,v,w\right)=C. \end{array}$$

Proved  □

**Theorem** **3.** *Let $Log_{i}C_{q}=\left\{\begin{array}{c} \sqrt{1-{\left(Log_{{}_{i}}E_{q}\right)}^{2}},Log_{{}_{i}}\left(\sqrt{1-F_{q}^{2}}\right),\\ Log_{{}_{i}}\left(\sqrt{1-G_{q}^{2}}\right) \end{array}\right\},\left(q=1,2\right)$ be any two LSHFNs with $0<i\leq \min\left\{E_{C},\sqrt{1-F_{C}^{2}},\sqrt{1-G_{C}^{2}}<1\right\}<1,i\neq 1$ then*

*(1) $Log_{{}_{i}}C_{1}⊕Log_{{}_{i}}C_{2}=Log_{{}_{i}}C_{2}⊕Log_{{}_{i}}C_{1};$*

*(2) $Log_{{}_{i}}C_{1}⊗Log_{{}_{i}}C_{2}=Log_{{}_{i}}C_{2}⊗Log_{{}_{i}}C_{1}.$*

**Proof.**  Straightforward.  □

**Theorem** **4.** *Let $Log_{i}C_{q}=\left\{\begin{array}{c} \sqrt{1-{\left(Log_{{}_{i}}E_{q}\right)}^{2}},Log_{{}_{i}}\left(\sqrt{1-F_{q}^{2}}\right),\\ Log_{{}_{i}}\left(\sqrt{1-G_{q}^{2}}\right) \end{array}\right\},$$\left(q=1,2,3\right)$ be any two LSHFNs with $0<i\leq \min\left\{E_{C},\sqrt{1-F_{C}^{2}},\sqrt{1-G_{C}^{2}}<1\right\}<1,$i≠1 then*

*(1) $\left(Log_{{}_{i}}C_{1}⊕Log_{{}_{i}}C_{2}\right)⊕Log_{{}_{i}}C_{3}=Log_{{}_{i}}C_{1}⊕\left(Log_{{}_{i}}C_{2}⊕Log_{{}_{i}}C_{3}\right),$*

*(2) $\left(Log_{{}_{i}}C_{1}⊕Log_{{}_{i}}C_{2}\right)⊗Log_{{}_{i}}C_{3}=Log_{{}_{i}}C_{1}⊗\left(Log_{{}_{i}}C_{2}⊗Log_{{}_{i}}C_{3}\right).$*

**Proof.**  Straightforward.  □

**Theorem** **5.** *Let $Log_{i}C_{q}=\left\{\begin{array}{c} \sqrt{1-{\left(Log_{{}_{i}}E_{q}\right)}^{2}},Log_{{}_{i}}\left(\sqrt{1-F_{q}^{2}}\right),\\ Log_{{}_{i}}\left(\sqrt{1-G_{q}^{2}}\right) \end{array}\right\}\left(q=1,2\right)$ be any two LSHFNs with $0<i\leq \min\left\{E_{C},\sqrt{1-F_{C}^{2}},\sqrt{1-G_{C}^{2}}<1\right\}<1,$i≠1,β,$\beta_{1},$$\beta_{2}>0$ are any real numbers. Then,*

*(1) $\beta \left(Log_{{}_{i}}C_{1}⊕Log_{{}_{i}}C_{2}\right)=\beta Log_{{}_{i}}C_{1}⊕\beta Log_{{}_{i}}C_{2};$*

*(2) ${\left(Log_{{}_{i}}C_{1}⊗Log_{{}_{i}}C_{2}\right)}^{\beta }={\left(Log_{{}_{i}}C_{1}\right)}^{\beta }⊗{\left(Log_{{}_{i}}C_{2}\right)}^{\beta };$*

*(3) $\beta Log_{{}_{i}}C_{1}⊕\beta Log_{{}_{i}}C_{1}=\left(\beta_{1}⊕\beta_{2}\right)Log_{{}_{i}}C_{1};$*

*(4) ${\left(Log_{{}_{i}}C_{1}\right)}^{\beta_{1}}⊗{\left(Log_{{}_{i}}C_{1}\right)}^{\beta_{2}}={\left(Log_{{}_{i}}C_{1}\right)}^{\beta_{1}+\beta_{2}};$*

*(5) ${\left({\left(Log_{{}_{i}}C_{1}\right)}^{\beta_{1}}\right)}^{\beta_{2}}={\left(Log_{{}_{i}}C_{1}\right)}^{\beta_{1}\beta_{2}}.$*

**Proof.**  $\left(1\right)$ As, from Definition 19, we have

$$Log_{{}_{i}}C_{1}⊕Log_{{}_{i}}C_{2}=\underset{\begin{array}{c} u_{q}\in \left(\sqrt{1-{\left(Log_{{}_{i}}E_{q}\right)}^{2}}\right),v_{q}\in Log_{{}_{i}}\left(\sqrt{1-F_{q}^{2}}\right),\\ w_{q}\in Log_{{}_{i}}\left(\sqrt{1-G_{q}^{2}}\right) \end{array}}{⋃}\left\{\begin{array}{c} \sqrt{1-{\left(Log_{{}_{i}}u_{1}\right)}^{2}{\left(Log_{{}_{i}}u_{2}\right)}^{2}},\\ Log_{{}_{i}}\sqrt{\left(1-v_{1}^{2}\right)}.Log_{{}_{i}}\sqrt{\left(1-v_{2}^{2}\right)},\\ Log_{{}_{i}}\sqrt{\left(1-w_{1}^{2}\right)}.Log_{{}_{i}}\sqrt{\left(1-w_{2}^{2}\right)} \end{array}\right\}$$

for any real number β>1,

$$\begin{array}{ccc} & & \beta \left(Log_{{}_{i}}C_{1}⊕Log_{{}_{i}}C_{2}\right)\\ & = & \;\underset{{}_{\begin{array}{c} u_{q}\in \left(\sqrt{1-{\left(Log_{{}_{i}}E_{q}\right)}^{2}}\right),v_{q}\in Log_{{}_{i}}\left(\sqrt{1-F_{q}^{2}}\right),\\ w_{q}\in Log_{{}_{i}}\left(\sqrt{1-G_{q}^{2}}\right) \end{array}}}{⋃}\left\{\begin{array}{c} \sqrt{1-{\left({\left(Log_{{}_{i}}u_{1}\right)}^{2}{\left(Log_{{}_{i}}u_{2}\right)}^{2}\right)}^{\beta }},\\ {\left(Log_{{}_{i}}\sqrt{\left(1-v_{1}^{2}\right)}.Log_{{}_{i}}\sqrt{\left(1-v_{2}^{2}\right)}\right)}^{\beta },\\ {\left(Log_{{}_{i}}\sqrt{\left(1-w_{1}^{2}\right)}.Log_{{}_{i}}\sqrt{\left(1-w_{2}^{2}\right)}\right)}^{\beta } \end{array}\right\}, \end{array}$$

$$\begin{array}{ccc} & = & \;\underset{\begin{array}{c} u_{1}\in \left(\sqrt{1-{\left(Log_{{}_{i}}E_{1}\right)}^{2}}\right),v_{1}\in Log_{{}_{i}}\left(\sqrt{1-F_{1}^{2}}\right),\\ w_{1}\in Log_{{}_{i}}\left(\sqrt{1-G_{1}^{2}}\right) \end{array}}{⋃}\left\{\begin{array}{c} \sqrt{1-{\left({\left(Log_{{}_{i}}u_{1}\right)}^{2}\right)}^{\beta }},\\ {\left(Log_{{}_{i}}\sqrt{\left(1-v_{1}^{2}\right)}\right)}^{\beta },\\ {\left(Log_{{}_{i}}\sqrt{\left(1-w_{1}^{2}\right)}\right)}^{\beta } \end{array}\right\}⊕\\ & & \;\underset{\begin{array}{c} u_{2}\in \left(\sqrt{1-{\left(Log_{{}_{i}}E_{2}\right)}^{2}}\right),v_{2}\in Log_{{}_{i}}\left(\sqrt{1-F_{2}^{2}}\right),\\ w_{2}\in Log_{{}_{i}}\left(\sqrt{1-G_{2}^{2}}\right) \end{array}}{⋃}\left\{\begin{array}{c} \sqrt{1-{\left({\left(Log_{{}_{i}}u_{2}\right)}^{2}\right)}^{\beta }},\\ {\left(Log_{{}_{i}}\sqrt{\left(1-v_{2}^{2}\right)}\right)}^{\beta },\\ {\left(Log_{{}_{i}}\sqrt{\left(1-w_{2}^{2}\right)}\right)}^{\beta } \end{array}\right\},\\ & = & \beta Log_{{}_{i}}C_{1}⊕\beta Log_{{}_{i}}C_{2}. \end{array}$$

$\left(2\right)$ We know from from def C,

$$\begin{array}{ccc} & & Log_{{}_{i}}C_{1}⊗Log_{{}_{i}}C_{2}\\ & = & \;\underset{\begin{array}{c} u_{q}\in \left(\sqrt{1-{\left(Log_{{}_{i}}E_{q}\right)}^{2}}\right),v_{q}\in Log_{{}_{i}}\left(\sqrt{1-F_{q}^{2}}\right),\\ w_{q}\in Log_{{}_{i}}\left(\sqrt{1-G_{q}^{2}}\right) \end{array}}{⋃}\;\left\{\begin{array}{c} \sqrt{1-{\left(Log_{{}_{i}}u_{1}\right)}^{2}}.\sqrt{1-{\left(Log_{{}_{i}}u_{2}\right)}^{2}},\\ \sqrt{\begin{array}{c} 1-{\left(1-Log_{{}_{i}}\left(\sqrt{1-v_{1}^{2}}\right)\right)}^{2}.\\ {\left(1-Log_{{}_{i}}\left(\sqrt{1-v_{2}^{2}}\right)\right)}^{2} \end{array}},\\ \sqrt{\begin{array}{c} 1-{\left(1-Log_{{}_{i}}\left(\sqrt{1-w_{1}^{2}}\right)\right)}^{2}.\\ {\left(1-Log_{{}_{i}}\left(\sqrt{1-w_{2}^{2}}\right)\right)}^{2} \end{array}} \end{array}\right\} \end{array}$$

for any real number β>0, we have

$$\begin{array}{ccc} & & {\left(Log_{{}_{i}}C_{1}⊗Log_{{}_{i}}C_{2}\right)}^{\beta }\\ & = & \underset{\begin{array}{c} u_{q}\in \left(\sqrt{1-{\left(Log_{{}_{i}}E_{q}\right)}^{2}}\right),v_{q}\in Log_{{}_{i}}\left(\sqrt{1-F_{q}^{2}}\right),\\ w_{q}\in Log_{{}_{i}}\left(\sqrt{1-G_{q}^{2}}\right) \end{array}}{⋃}\;\left\{\begin{array}{c} {\left(\sqrt{1-{\left(Log_{{}_{i}}u_{1}\right)}^{2}}\right)}^{\beta }.{\left(\sqrt{1-{\left(Log_{{}_{i}}u_{2}\right)}^{2}}\right)}^{\beta },\\ \sqrt{\begin{array}{c} 1-{\left({\left(1-Log_{{}_{i}}\left(\sqrt{1-v_{1}^{2}}\right)\right)}^{2}\right)}^{\beta }.\\ {\left({\left(1-Log_{{}_{i}}\left(\sqrt{1-v_{2}^{2}}\right)\right)}^{2}\right)}^{\beta } \end{array}},\\ \sqrt{\begin{array}{c} 1-{\left({\left(1-Log_{{}_{i}}\left(\sqrt{1-w_{1}^{2}}\right)\right)}^{2}\right)}^{\beta }.\\ {\left({\left(1-Log_{{}_{i}}\left(\sqrt{1-w_{2}^{2}}\right)\right)}^{2}\right)}^{\beta } \end{array}} \end{array}\right\}\\ & = & {\left(Log_{{}_{i}}C_{1}\right)}^{\beta }⊗{\left(Log_{{}_{i}}C_{2}\right)}^{\beta }. \end{array}$$

(3–5) can be proven in a similar way.  □

**Definition** **20.** *Suppose the LSHFN $\;Log_{i}C_{q}=\left\{\begin{array}{c} \sqrt{1-{\left(Log_{{}_{i}}E_{q}\right)}^{2}},\\ Log_{{}_{i}}\left(\sqrt{1-F_{q}^{2}}\right),\\ Log_{{}_{i}}\left(\sqrt{1-G_{q}^{2}}\right) \end{array}\right\},$ then the score and accuracy functions are described as below,*

(14) $$\begin{array}{ccc} S\left(Log_{i}C_{q}\right) & = & \frac{1}{l_{E_{q}}\left(C\right)}\sum \left(1-{\left(Log_{{}_{i}}E_{q}\right)}^{2}\right)-\frac{1}{l_{F_{q}}\left(C\right)}\sum {\left(Log_{{}_{i}}\sqrt{1-F_{q}^{2}}\right)}^{2}\\ & & -\frac{1}{l_{G_{q}}\left(C\right)}\sum {\left(Log_{{}_{i}}\sqrt{1-G_{q}^{2}}\right)}^{2} \end{array}$$

*and an accuracy function H is described as*

(15) $$\begin{array}{ccc} S\left(Log_{i}C_{q}\right) & = & \frac{1}{l_{E_{q}}\left(C\right)}\sum \left(1-{\left(Log_{{}_{i}}E_{q}\right)}^{2}\right)+\frac{1}{l_{F_{q}}\left(C\right)}\sum {\left(Log_{{}_{i}}\sqrt{1-F_{q}^{2}}\right)}^{2}\\ & & +\frac{1}{l_{G_{q}}\left(C\right)}\sum {\left(Log_{{}_{i}}\sqrt{1-G_{q}^{2}}\right)}^{2}. \end{array}$$

On the basis of score and accuracy functions, a comparison system is described as

**Definition** **21.** *Let $Log_{i}C_{q}=\left\{\begin{array}{c} \sqrt{1-{\left(Log_{{}_{i}}E_{q}\right)}^{2}},\\ Log_{{}_{i}}\left(\sqrt{1-F_{q}^{2}}\right),\\ Log_{{}_{i}}\left(\sqrt{1-G_{q}^{2}}\right) \end{array}\right\},$$\left(q=1,2\right)$ be any two LSHFNs. Then, the comparison procedure is given as*

*(1) If $S\left(Log_{i}C_{1}\right)>S\left(Log_{i}C_{2}\right)$, then $Log_{i}C_{1}>Log_{i}C_{2}$;*

*(2) If $S\left(Log_{i}C_{1}\right)=S\left(Log_{i}C_{2}\right)$ and $H\left(Log_{i}C_{1}\right)>H\left(Log_{i}C_{2}\right)$, then $Log_{i}C_{1}>Log_{i}C_{2}$;*

*(3) If $S\left(Log_{i}C_{1}\right)=S\left(Log_{i}C_{2}\right)$ and $H\left(Log_{i}C_{1}\right)=H\left(Log_{i}C_{2}\right)$, then $Log_{i}C_{1}=Log_{i}C_{2}$.*

## 4. Logarithmic Spherical Hesitant Fuzzy Aggregation Operators

Here, we express certain logarithmic aggregation operators, namely logarithmic spherical hesitant fuzzy weighted averaging/geometric aggregation operators. Furthermore, their features have been conferred in detail.

### 4.1. Logarithmic Averaging Aggregation Operators

**Definition** **22.** *Suppose $C_{q}=\left\{E_{q},F_{q},G_{q}\right\}\left(q=1,2\ldots m\right)$ be the set of SHFN. If*

$$0<i\leq \min\left\{E_{C},\sqrt{1-F_{C}^{2}},\sqrt{1-G_{C}^{2}}<1\right\}<1,i\neq 1.$$

*Then, Log−SHFWA is described as*

(16) $$Log-SHFWA\left(C_{1,}C_{2}\ldots C_{m}\right)=\sum_{q=1}^{m}\beta_{{}_{q}}.Log_{i_{q}}C_{q}$$

*where $\beta_{{}_{q}}=\left(q=1,2\ldots m\right)$ is the weight vector of Log-SHFWA such that $\beta_{q}\in \left[0,1\right]$ and $\sum_{q=1}^{m}\beta_{q}=1.$*

**Theorem** **6.** *Suppose $C_{q}=\left\{E_{q},F_{q},G_{q}\right\}\left(q=1,2\ldots m\right)$ be the set of SHFN. If*

$$0<i_{q}\leq \min\left\{E_{C},\sqrt{1-F_{C}^{2}},\sqrt{1-G_{C}^{2}}<1\right\}<1,i\neq 1.$$

*Then*

$$Log-SHFWA\left(C_{1,}C_{2}\ldots C_{m}\right)=\left\{\begin{array}{c} \sqrt{1-\prod_{q=1}^{m}{\left(Log_{i_{q}}u_{q}\right)}^{2\beta_{q}}},\\ \prod_{q=1}^{m}{\left(Log_{i_{q}}\left(\sqrt{1-v_{q}^{2}}\right)\right)}^{\beta_{q}},\\ \prod_{q=1}^{m}{\left(Log_{i_{q}}\left(\sqrt{1-w_{q}^{2}}\right)\right)}^{\beta_{q}} \end{array}\right\}$$

*where $\beta_{{}_{q}}=\left(q=1,2\ldots m\right)$ is the weight vector of Log-SHFWA such that $\beta_{q}\in \left[0,1\right]$ and $\sum_{q=1}^{m}\beta_{q}=1.$*

**Proof.**  To prove the given result, we use the principal of mathematical induction.

**Step-1:** For q=2,

$$Log-SHFWA\left(C_{1,}C_{2}\right)=\beta_{{}_{1}}.Log_{i_{1}}C_{1}⊕\beta_{{}_{2}}.Log_{i_{2}}C_{2}$$

where

$$\begin{array}{ccc} & & \beta_{{}_{1}}Log_{i_{1}}C_{1}\\ & = & \underset{\left(u_{1},v_{1},w_{1}\right)\in \left(\begin{array}{c} Log_{i_{1}}E_{1},Log_{i_{1}}\left(\sqrt{1-F_{1}^{2}}\right),\\ Log_{i_{1}}\left(\sqrt{1-G_{1}^{2}}\right) \end{array}\right)}{⋃}\left\{\begin{array}{c} \sqrt{1-{\left({\left(Log_{i_{1}}u_{1}\right)}^{2}\right)}^{\beta_{{}_{1}}}},\\ {\left(Log_{i_{1}}\left(\sqrt{1-v_{1}^{2}}\right)\right)}^{\beta_{q}},\\ {\left(Log_{i_{1}}\left(\sqrt{1-w_{1}^{2}}\right)\right)}^{\beta_{q}} \end{array}\right\}; \end{array}$$

and

$$\begin{array}{ccc} & & \beta_{{}_{2}}Log_{i_{2}}C_{2}\\ & = & \underset{\left(u_{2},v_{2},w_{2}\right)\in \left(\begin{array}{c} Log_{i_{2}}E_{2},Log_{i_{2}}\left(\sqrt{1-F_{2}^{2}}\right),\\ Log_{i_{2}}\left(\sqrt{1-G_{2}^{2}}\right) \end{array}\right)}{⋃}\left\{\begin{array}{c} \sqrt{1-{\left({\left(Log_{i_{2}}u_{2}\right)}^{2}\right)}^{\beta_{{}_{2}}}},\\ {\left(Log_{i_{2}}\left(\sqrt{1-v_{2}^{2}}\right)\right)}^{\beta_{2}},\\ {\left(Log_{i_{2}}\left(\sqrt{1-w_{2}^{2}}\right)\right)}^{\beta_{2}} \end{array}\right\}; \end{array}$$

$$\begin{array}{ccc} & & Log-SHFWA\left(C_{1,}C_{2}\right)\\ & = & \underset{\left(u_{1},v_{1},w_{1}\right)\in \left(\begin{array}{c} Log_{i_{1}}E_{1},Log_{i_{1}}\left(\sqrt{1-F_{1}^{2}}\right),\\ Log_{i_{1}}\left(\sqrt{1-G_{1}^{2}}\right) \end{array}\right)}{⋃}\left\{\begin{array}{c} \sqrt{1-{\left({\left(Log_{i_{1}}u_{1}\right)}^{2}\right)}^{\beta_{{}_{1}}}},\\ {\left(Log_{i_{1}}\left(\sqrt{1-v_{1}^{2}}\right)\right)}^{\beta_{1}},\\ {\left(Log_{i_{1}}\left(\sqrt{1-w_{1}^{2}}\right)\right)}^{\beta_{1}} \end{array}\right\}⊕\\ & & \underset{\left(u_{2},v_{2},w_{2}\right)\in \left(\begin{array}{c} Log_{i_{2}}E_{2},Log_{i_{2}}\left(\sqrt{1-F_{2}^{2}}\right),\\ Log_{i_{2}}\left(\sqrt{1-G_{2}^{2}}\right) \end{array}\right)}{⋃}\left\{\begin{array}{c} \sqrt{1-{\left({\left(Log_{i_{2}}u_{2}\right)}^{2}\right)}^{\beta_{{}_{2}}}},\\ {\left(Log_{i_{2}}\left(\sqrt{1-v_{2}^{2}}\right)\right)}^{\beta_{2}},\\ {\left(Log_{i_{2}}\left(\sqrt{1-w_{2}^{2}}\right)\right)}^{\beta_{2}} \end{array}\right\} \end{array}$$

By using Definition 19, we get

$$\begin{array}{ccc} & & Log-SHFWA\left(C_{1,}C_{2}\right)\\ & = & \underset{\left(u_{q},v_{q},w_{q}\right)\in \left(\begin{array}{c} Log_{i_{q}}E_{q},Log_{i_{q}}\left(\sqrt{1-F_{q}^{2}}\right),\\ Log_{i_{q}}\left(\sqrt{1-G_{q}^{2}}\right) \end{array}\right)}{⋃}\left\{\begin{array}{c} \sqrt{1-{\left({\left(Log_{i_{1}}u_{1}\right)}^{2}\right)}^{\beta_{{}_{1}}}.{\left({\left(Log_{i_{2}}u_{2}\right)}^{2}\right)}^{{}^{\beta_{2}}}},\\ {\left(Log_{i_{1}}\left(\sqrt{1-v_{1}^{2}}\right)\right)}^{\beta_{1}}.{\left(Log_{i_{2}}\left(\sqrt{1-v_{2}^{2}}\right)\right)}^{\beta_{2}},\\ {\left(Log_{i_{1}}\left(\sqrt{1-w_{1}^{2}}\right)\right)}^{\beta_{1}}.{\left(Log_{i_{2}}\left(\sqrt{1-w_{2}^{2}}\right)\right)}^{\beta_{2}} \end{array}\right\}. \end{array}$$

$$\begin{array}{ccc} & & Log-SHFWA\left(C_{1,}C_{2}\right)\\ & = & \underset{\left(u_{q},v_{q},w_{q}\right)\in \left(\begin{array}{c} Log_{i_{q}}E_{q},Log_{i_{q}}\left(\sqrt{1-F_{q}^{2}}\right),\\ Log_{i_{q}}\left(\sqrt{1-G_{q}^{2}}\right) \end{array}\right)}{⋃}\left\{\begin{array}{c} \sqrt{1-\prod_{q=1}^{2}{\left(Log_{i_{q}}u_{q}\right)}^{2\beta_{q}}},\\ \prod_{q=1}^{2}{\left(Log_{i_{q}}\left(\sqrt{1-v_{q}^{2}}\right)\right)}^{\beta_{q}},\\ \prod_{q=1}^{2}{\left(Log_{i_{q}}\left(\sqrt{1-w_{q}^{2}}\right)\right)}^{\beta_{q}} \end{array}\right\}. \end{array}$$

**Step-2:** Suppose that given result is true for q=x, i.e.,

$$\begin{array}{ccc} & & Log-SHFWA\left(C_{1,}C_{2},\ldots C_{x}\right)\\ & = & \underset{\left(u_{q},v_{q},w_{q}\right)\in \left(\begin{array}{c} Log_{i_{q}},Log_{i_{q}}\left(\sqrt{1-F_{q}^{2}}\right),\\ Log_{i_{q}}\left(\sqrt{1-G_{q}^{2}}\right) \end{array}\right)}{⋃}\;\left\{\begin{array}{c} \sqrt{1-\prod_{q=1}^{x}{\left(Log_{i_{q}}u_{q})\right)}^{2\beta_{q}}},\\ \prod_{q=1}^{x}{\left(Log_{i_{q}}\left(\sqrt{1-v_{q}^{2}}\right)\right)}^{\beta_{q}},\\ \prod_{q=1}^{x}{\left(Log_{i_{q}}\left(\sqrt{1-w_{q}^{2}}\right)\right)}^{\beta_{q}} \end{array}\right\}; \end{array}$$

**Step-3:** Now, we have to prove that result is true for q=x+1.

$$Log-SHFWA\left(C_{1,}C_{2},\ldots C_{x+1}\right)=\sum_{q=1}^{m}\beta_{{}_{q}}Log_{i_{q}}C_{q}+\beta_{{}_{m+1}}Log_{i_{m+1}}C_{m+1}$$

$$\begin{array}{ccc} & & Log-SHFWA\left(C_{1,}C_{2},\ldots C_{x+1}\right)\\ & = & \underset{\left(u_{q},v_{q},w_{q}\right)\in \left(\begin{array}{c} Log_{i_{q}}E_{q},Log_{i_{q}}\left(\sqrt{1-F_{q}^{2}}\right),\\ Log_{i_{q}}\left(\sqrt{1-G_{q}^{2}}\right) \end{array}\right)}{⋃}\;\left\{\begin{array}{c} \sqrt{1-\prod_{q=1}^{x}{\left(Log_{i_{q}}u_{q})\right)}^{2\beta_{q}}},\\ \prod_{q=1}^{x}{\left(Log_{i_{q}}\left(\sqrt{1-v_{q}^{2}}\right)\right)}^{\beta_{q}},\\ \prod_{q=1}^{x}{\left(Log_{i_{q}}\left(\sqrt{1-w_{q}^{2}}\right)\right)}^{\beta_{q}} \end{array}\right\}⊕\\ & & \underset{\left(u_{x+1},v_{x+1},w_{x+1}\right)\in \left(\begin{array}{c} Log_{i_{x+1}}E_{x+1},Log_{i_{x+1}}\left(\sqrt{1-F_{x+1}^{2}}\right),\\ Log_{i_{x+1}}\left(\sqrt{1-G_{x+1}^{2}}\right) \end{array}\right)}{⋃}\left\{\begin{array}{c} \sqrt{1-{\left(Log_{i_{q}}u_{x+1}\right)}^{2\beta_{x+1}}},\\ {\left(Log_{i_{q}}\sqrt{1-v_{x+1}^{2}}\right)}^{\beta_{x+1}},\\ {\left(Log_{i_{q}}\sqrt{1-w_{x+1}^{2}}\right)}^{\beta_{x+1}} \end{array}\right\}\\ & = & \underset{\left(u_{q},v_{q},w_{q}\right)\in \left(\begin{array}{c} Log_{i_{q}}E_{q},Log_{i_{q}}\left(\sqrt{1-F_{q}^{2}}\right),\\ Log_{i_{q}}\left(\sqrt{1-G_{q}^{2}}\right) \end{array}\right)}{⋃}\;\left\{\begin{array}{c} \sqrt{1-\prod_{q=1}^{x+1}{\left(Log_{i_{q}}u_{q})\right)}^{2\beta_{q}}},\\ \prod_{q=1}^{x+1}{\left(Log_{i_{q}}\left(\sqrt{1-v_{q}^{2}}\right)\right)}^{\beta_{q}},\\ \prod_{q=1}^{x+1}{\left(Log_{i_{q}}\left(\sqrt{1-w_{q}^{2}}\right)\right)}^{\beta_{q}} \end{array}\right\} \end{array}$$

Therefore, the given result is true for all positive integers, i.e.,

$$\begin{array}{ccc} & & Log-SHFWA\left(C_{1,}C_{2}\ldots C_{m}\right)\\ & = & \underset{\left(u_{q},v_{q},w_{q}\right)\in \left(\begin{array}{c} Log_{i_{q}}E_{q},Log_{i_{q}}\left(\sqrt{1-F_{q}^{2}}\right),\\ Log_{i_{q}}\left(\sqrt{1-G_{q}^{2}}\right) \end{array}\right)}{⋃}\;\;\left\{\begin{array}{c} \sqrt{1-\prod_{q=1}^{m}{\left(Log_{i_{q}}u_{q})\right)}^{2\beta_{q}}},\\ \prod_{q=1}^{m}{\left(Log_{i_{q}}\left(\sqrt{1-v_{q}^{2}}\right)\right)}^{\beta_{q}},\\ \prod_{q=1}^{m}{\left(Log_{i_{q}}\left(\sqrt{1-w_{q}^{2}}\right)\right)}^{\beta_{q}} \end{array}\right\}. \end{array}$$

Similarly, if

$$0<\frac{1}{i_{q}}\leq \min\left\{E_{C},\sqrt{1-F_{C}^{2}},\sqrt{1-G_{C}^{2}}<1\right\}<1,i\neq 1.$$

We can prove

$$\begin{array}{ccc} & & Log-SHFWA\left(C_{1,}C_{2}\ldots C_{m}\right)\\ & = & \underset{\left(u_{q},v_{q},w_{q}\right)\in \left(\begin{array}{c} Log_{\frac{1}{i_{q}}}E_{q},Log_{\frac{1}{i_{q}}}\left(\sqrt{1-F_{q}^{2}}\right),\\ Log_{\frac{1}{i_{q}}}\left(\sqrt{1-G_{q}^{2}}\right) \end{array}\right)}{⋃}\;\;\left\{\begin{array}{c} \sqrt{1-\prod_{q=1}^{m}{\left(Log_{\frac{1}{i_{q}}}u_{q})\right)}^{2\beta_{q}}},\\ \prod_{q=1}^{m}{\left(Log_{\frac{1}{i_{q}}}\left(\sqrt{1-v_{q}^{2}}\right)\right)}^{\beta_{q}},\\ \prod_{q=1}^{m}{\left(Log_{\frac{1}{i_{q}}}\left(\sqrt{1-w_{q}^{2}}\right)\right)}^{\beta_{q}} \end{array}\right\}. \end{array}$$

Proved.  □

**Remark** **1.** *If $i_{1}=i_{2}=i_{3}=\ldots i_{m}=i$, that is,*

$$0<i\leq \min\left\{E_{C},\sqrt{1-F_{C}^{2}},\sqrt{1-G_{C}^{2}}<1\right\}<1,i\neq 1,$$

*then the Log−SHFWA operator can be reduced as below,*

$$\begin{array}{ccc} & & Log-SHFWA\left(C_{1,}C_{2}\ldots C_{m}\right)\\ & = & \underset{\left(u_{q},v_{q},w_{q}\right)\in \left(\begin{array}{c} Log_{i}E_{q},Log_{i}\left(\sqrt{1-F_{q}^{2}}\right),\\ Log_{i}\left(\sqrt{1-G_{q}^{2}}\right) \end{array}\right)}{⋃}\left\{\begin{array}{c} \sqrt{1-\prod_{q=1}^{m}{\left(Log_{i}u_{q})\right)}^{2\beta_{q}}},\\ \prod_{q=1}^{m}{\left(Log_{i}\left(\sqrt{1-v_{q}^{2}}\right)\right)}^{\beta_{q}},\\ \prod_{q=1}^{m}{\left(Log_{i}\left(\sqrt{1-w_{q}^{2}}\right)\right)}^{\beta_{q}} \end{array}\right\}. \end{array}$$

The following properties are satisfied by the Log−SHFWA:

(1) **Idempotency**: Let $C_{q}=\left\{E_{q},F_{q},G_{q}\right\}\left(q=1,2\ldots m\right)$ be the set of SHFN. if all $\;C_{q}=C=\left\{u,v,w\right\},\left(q\in N\right)$ Then,

$$Log-SHFWA\left(C_{1,}C_{2}\ldots C_{m}\right)=C$$

(2) **Boundedness**: Let $C_{q}=\left\{E_{q},F_{q},G_{q}\right\}\in SHFS$ $\left(q\in N\right).$ If $C_{q}^{-}=\left\{\min\left(E_{q}\right)\right\}$, $\left\{\min\left(F_{q}\right),\max\left(G_{q}\right)\right\}$ and $C_{q}^{+}=\left\{\max\left(E_{q}\right),\min\left(F_{q}\right),\min\left(G_{q}\right)\right\},$ then we have

$$C_{q}^{-}\subseteq Log-SHFWA\left(C_{1,}C_{2}\ldots C_{m}\right)\subseteq C_{q}^{+}.$$

(3) **Monotonicity**: Let $C_{q}=\left\{E_{q},F_{q},G_{q}\right\}$&$C_{q}^{*}=\left\{E_{q}^{*},F_{q}^{*},G_{q}^{*}\right\}$ ∈SHFS $\left(q\in N\right).$ If $C_{q}\subseteq C_{q}^{*}$, then

$$Log-SHFWA\left(C_{1,}C_{2}\ldots C_{m}\right)\subseteq Log-SHFWA\left(C_{1}^{*},C_{2}^{*}\ldots C_{m}^{*}\right).$$

**Definition** **23.** *Let $C_{q}=\left\{E_{q},F_{q},G_{q}\right\}\left(q=1,2\ldots m\right)$ be the set of SHFN. If $0<i\leq \min\left\{\begin{array}{c} E_{C},\\ \sqrt{1-F_{C}^{2}},\\ \sqrt{1-G_{C}^{2}} \end{array}\right\}<1,i\neq 1.$ Then, Log−SHFOWA is described as*

$$\begin{array}{ccc} Log-SHFOWA\left(C_{1,}C_{2}\ldots C_{m}\right) & = & \beta_{1}C_{\ell \left(1\right)}⊕\beta_{2}C_{\ell \left(2\right)}⊕\ldots ⊕\beta_{m}C_{\ell \left(q\right)}\\ & = & \sum_{q=1}^{m}\beta_{{}_{q}}.Log_{i_{q}}C_{\ell \left(q\right)} \end{array}$$

*where $\beta_{{}_{q}}=\left(q=1,2\ldots m\right)$ is weight information with $\beta_{q}\geq 0$, $\sum_{q=1}^{m}\beta_{q}=1$ and the qth biggest weighted value is $C_{\ell \left(q\right)}$ so by total order $C_{\ell \left(1\right)}\geq C_{\ell \left(2\right)}\geq \ldots \geq C_{\ell \left(m\right)}.$*

**Theorem** **7.** *Let $C_{q}=\left\{E_{q},F_{q},G_{q}\right\}\left(q=1,2\ldots m\right)$ be the set of SHFN. If*

$$0<i\leq \min\left\{E_{C},\sqrt{1-F_{C}^{2}},\sqrt{1-G_{C}^{2}}<1\right\}<1,i\neq 1.$$

*Then,*

$$\begin{array}{ccc} & & Log-SHFOWA\left(C_{1,}C_{2}\ldots C_{m}\right)\\ & = & \underset{\left(u_{q},v_{q},w_{q}\right)\in \left(\begin{array}{c} Log_{{}_{i}}E_{q},Log_{{}_{i}}\left(\sqrt{1-F_{q}^{2}}\right),\\ Log_{{}_{i}}\left(\sqrt{1-G_{q}^{2}}\right) \end{array}\right)}{⋃}\left\{\begin{array}{c} \sqrt{1-\prod_{q=1}^{m}{\left(Log_{{}_{i}}E_{\ell \left(q\right)}\right)}^{2\beta_{\ell \left(q\right)}}},\\ \prod_{q=1}^{m}{\left(Log_{i_{q}}\left(\sqrt{1-F_{\ell \left(q\right)}^{2}}\right)\right)}^{\beta_{\ell \left(q\right)}},\\ \prod_{q=1}^{m}{\left(Log_{i_{q}}\left(\sqrt{1-F_{\ell \left(q\right)}^{2}}\right)\right)}^{\beta_{\ell \left(q\right)}} \end{array}\right\}. \end{array}$$

*where $\beta_{{}_{q}}=\left(q=1,2\ldots m\right)$ is weight information with $\beta_{q}\geq 0$, $\sum_{q=1}^{m}\beta_{q}=1$ and the qth biggest weighted value is $C_{\ell \left(q\right)}$ so by total order $C_{\ell \left(1\right)}\geq C_{\ell \left(2\right)}\geq \ldots \geq C_{\ell \left(m\right)}.$*

**Proof.**  The proof follows from Theorem 6.  □

The following properties are satisfied by the Log−SHFOWA:

(1) **Idempotency**: Let $C_{q}=\left\{E_{q},F_{q},G_{q}\right\}\left(q=1,2\ldots m\right)$ be the set of SHFN. If all $\;C_{q}=C=\left\{u,v,w\right\},\left(q\in N\right)$ Then,

$$Log-SHFOWA\left(C_{1,}C_{2}\ldots C_{m}\right)=C$$

(2) **Boundedness**: Let $C_{q}=\left\{E_{q},F_{q},G_{q}\right\}\in SHFS$ $\left(q\in N\right).$ If $C_{q}^{-}=\left\{\min\left(E_{q}\right),\min\left(F_{q}\right),\max\left(G_{q}\right)\right\}$ and $C_{q}^{+}=\left\{\max\left(E_{q}\right),\min\left(F_{q}\right),\min\left(G_{q}\right)\right\},$ then we have

$$C_{q}^{-}\subseteq Log-SHFOWA\left(C_{1,}C_{2}\ldots C_{m}\right)\subseteq C_{q}^{+}.$$

(3) **Monotonicity**: Let $C_{q}=\left\{E_{q},F_{q},G_{q}\right\}$&$C_{q}^{*}=\left\{E_{q}^{*},F_{q}^{*},G_{q}^{*}\right\}$ ∈SHFS $\left(q\in N\right).$ If $C_{q}\subseteq C_{q}^{*}$, then

$$Log-SHFOWA\left(C_{1,}C_{2}\ldots C_{m}\right)\subseteq Log-SHFOWA\left(C_{1}^{*},C_{2}^{*}\ldots C_{m}^{*}\right).$$

### 4.2. Logarithmic Geometric Aggregation Operators

**Definition** **24.** *Let $C_{q}=\left\{E_{q},F_{q},G_{q}\right\}\in SHFS\left(q\in N\right).$ Then, weighted geometric can be defined as in the following:*

$$\begin{array}{ccc} Log-SHFWG\left(C_{1,}C_{2}\ldots C_{m}\right) & = & {\left(C_{1}\right)}^{\beta_{1}}⊗{\left(C_{2}\right)}^{\beta_{2}}⊗\ldots ⊗{\left(C_{n}\right)}^{\beta_{m}}\\ & = & \prod_{q=1}^{m}{\left(Log_{i_{q}}C_{q}\right)}^{\beta_{q}} \end{array}$$

*where ${\left(\beta_{1},\beta_{2},\ldots \beta_{m}\right)}^{T}$ is weight information of $\left(C_{1,}C_{2}\ldots C_{m}\right)$ such that $\beta_{q}\geq 0$; $\sum_{q=1}^{m}\beta_{q}=1.$*

**Theorem** **8.** *Suppose $C_{q}=\left\{E_{q},F_{q},G_{q}\right\}\left(q=1,2\ldots m\right)$ be the set of SHFN. If*

$$0<i_{q}\leq \min\left\{E_{C},\sqrt{1-F_{C}^{2}},\sqrt{1-G_{C}^{2}}<1\right\}<1,i\neq 1.$$

*Then,*

$$\begin{array}{ccc} & & Log-SHFWG\left(C_{1,}C_{2}\ldots C_{m}\right)\\ & = & \underset{\left(u_{q},v_{q},w_{q}\right)\in \left(\begin{array}{c} 1-Log_{{}_{i}}E_{q},Log_{{}_{i}}\left(1-F_{q}\right),\\ Log_{{}_{i}}\left(1-G_{q}\right) \end{array}\right)}{⋃}\left\{\begin{array}{c} {\left(\prod_{q=1}^{m}\sqrt{1-{\left(Log_{{}_{i}}u_{q}\right)}^{2}}\right)}^{\beta },\\ \sqrt{1-{\left(1-\prod_{q=1}^{m}{\left(Log_{{}_{i}}\sqrt{1-v_{q}^{2}}\right)}^{2}\right)}^{\beta }},\\ \sqrt{1-{\left(1-\prod_{q=1}^{m}{\left(Log_{{}_{i}}\sqrt{1-w_{q}^{2}}\right)}^{2}\right)}^{\beta }} \end{array}\right\}. \end{array}$$

**Proof.**  The proof follows from Theorem 6.  □

The following properties are satisfied by the Log−SHFWG:

(1) **Idempotency**: Let $C_{q}=\left\{E_{q},F_{q},G_{q}\right\}\left(q=1,2\ldots m\right)$ be the set of SHFN. if all $\;C_{q}=C=\left\{u,v,w\right\},\left(q\in N\right)$ Then,

$$Log-SHFWG\left(C_{1,}C_{2}\ldots C_{m}\right)=C$$

(2) **Boundedness**: Let $C_{q}=\left\{E_{q},F_{q},G_{q}\right\}\in SHFS$ $\left(q\in N\right).$ If $C_{q}^{-}=\left\{\min\left(E_{q}\right),\min\left(F_{q}\right),\max\left(G_{q}\right)\right\}$ and $C_{q}^{+}=\left\{\max\left(E_{q}\right),\min\left(F_{q}\right),\min\left(G_{q}\right)\right\},$ then we have

$$C_{q}^{-}\subseteq Log-SHFWG\left(C_{1,}C_{2}\ldots C_{m}\right)\subseteq C_{q}^{+}.$$

(3) **Monotonicity**: Let $C_{q}=\left\{E_{q},F_{q},G_{q}\right\}$&$C_{q}^{*}=\left\{E_{q}^{*},F_{q}^{*},G_{q}^{*}\right\}$ ∈SHFS $\left(q\in N\right).$ If $C_{q}\subseteq C_{q}^{*}$, then

$$Log-SHFWG\left(C_{1,}C_{2}\ldots C_{m}\right)\subseteq Log-SHFWG\left(C_{1}^{*},C_{2}^{*}\ldots C_{m}^{*}\right).$$

**Definition** **25.** *Let $C_{q}=\left\{E_{q},F_{q},G_{q}\right\}\in SHFS\left(q\in N\right).$ Then, the logarithmic spherical hesitant ordered weighted geometric operator is described as*

$$\begin{array}{ccc} & & Log-SHFOWG\left(C_{1,}C_{2}\ldots C_{m}\right)={\left(C_{\ell \left(1\right)}\right)}^{\beta_{1}}⊗{\left(C_{\ell \left(2\right)}\right)}^{\beta_{2}}⊗\ldots ⊗{\left(C_{\ell \left(n\right)}\right)}^{\beta_{m}}\\ & = & \prod_{q=1}^{m}{\left(Log_{i_{q}}C_{\ell \left(q\right)}\right)}^{\beta_{q}} \end{array}$$

*where $\ell \left(q\right)$ denote the order according to $\left(\ell \left(1\right),\ell \left(2\right),\ell \left(3\right),\ldots ,\ell \left(m\right)\right)$ and ${\left(\beta_{1},\beta_{2},\ldots \beta_{m}\right)}^{T}$ is weight information of $\left(C_{1,}C_{2}\ldots C_{m}\right)$ such that $\beta_{q}\geq 0$; $\sum_{q=1}^{m}\beta_{q}=1.$*

**Theorem** **9.** *Suppose $C_{q}=\left\{E_{q},F_{q},G_{q}\right\}\left(q=1,2\ldots m\right)$ be the set of SHFN. If*

$$0<i_{q}\leq \min\left\{E_{C},\sqrt{1-F_{C}^{2}},\sqrt{1-G_{C}^{2}}<1\right\}<1,i\neq 1.$$

*Then*

$$\begin{array}{ccc} & & Log-SHFOWG\left(C_{1,}C_{2}\ldots C_{m}\right)\\ & = & \underset{\left(u_{q},v_{q},w_{q}\right)\in \left(\begin{array}{c} 1-Log_{{}_{i}}E_{q},Log_{{}_{i}}\left(1-F_{q}\right),\\ Log_{{}_{i}}\left(1-G_{q}\right) \end{array}\right)}{⋃}\left\{\begin{array}{c} {\left(\prod_{q=1}^{m}\sqrt{1-{\left(Log_{{}_{i}}u_{\ell \left(q\right)}\right)}^{2}}\right)}^{\beta },\\ \sqrt{1-{\left(1-\prod_{q=1}^{m}{\left(Log_{{}_{i}}\sqrt{1-v_{\ell \left(q\right)}^{2}}\right)}^{2}\right)}^{\beta }},\\ \sqrt{1-{\left(1-\prod_{q=1}^{m}{\left(Log_{{}_{i}}\sqrt{1-w_{\ell \left(q\right)}^{2}}\right)}^{2}\right)}^{\beta }} \end{array}\right\}. \end{array}$$

**Proof.**  Prove is follow from Theorem 6.  □

The following properties are satisfied by the Log−SHFOWG:

(1) **Idempotency**: Let $C_{q}=\left\{E_{q},F_{q},G_{q}\right\}\left(q=1,2\ldots m\right)$ be the set of SHFN. if all $\;C_{q}=C=\left\{u,v,w\right\},\left(q\in N\right)$ Then,

$$Log-SHFOWG\left(C_{1,}C_{2}\ldots C_{m}\right)=C$$

(2) **Boundedness**: Let $C_{q}=\left\{E_{q},F_{q},G_{q}\right\}\in SHFS$ $\left(q\in N\right).$ If $C_{q}^{-}=\left\{\min\left(E_{q}\right),\min\left(F_{q}\right),\max\left(G_{q}\right)\right\}$ and $C_{q}^{+}=\left\{\max\left(E_{q}\right),\min\left(F_{q}\right),\min\left(G_{q}\right)\right\},$ then we have

$$C_{q}^{-}\subseteq Log-SHFOWG\left(C_{1,}C_{2}\ldots C_{m}\right)\subseteq C_{q}^{+}.$$

(3) **Monotonicity**: Let $C_{q}=\left\{E_{q},F_{q},G_{q}\right\}$&$C_{q}^{*}=\left\{E_{q}^{*},F_{q}^{*},G_{q}^{*}\right\}$ ∈SHFS $\left(q\in N\right).$ If $C_{q}\subseteq C_{q}^{*}$, then

$$Log-SHFOWG\left(C_{1,}C_{2}\ldots C_{m}\right)\subseteq Log-SHFOWG\left(C_{1}^{*},C_{2}^{*}\ldots C_{m}^{*}\right).$$

## 5. Algorithm for Decision Making Problems

Here, we have established a framework for addressing improbability/uncertainty in decision-making (DM) under spherical hesitant fuzzy information. Consider a DM problem with a set of m alternatives $\left\{A_{1},A_{2},\ldots \ldots ,A_{g}\right\}$ and $\left\{B_{1},B_{2},\ldots .,B_{h}\right\}$ be a set of attributes with weights ${\left(\beta_{1},\beta_{2},\ldots \beta_{m}\right)}^{T}$ such that $\beta_{q}\in \left[0,1\right]$, $\sum_{q=1}^{m}\beta_{q}=1.$ To assess the performance of qth alternative $A_{q}$ under the qth attribute $B_{q},$ let $\left\{{\overset{˚}{D}}_{1},{\overset{˚}{D}}_{2},\ldots .,{\overset{˚}{D}}_{\hat{j}}\right\}$ be a set of decision-makers (DMs) and ${\left(\eta_{1},\eta_{2},\ldots .,\eta_{\hat{j}}\right)}^{T}$ be DMs weights such that $\eta_{s}\in \left[0,1\right]$, $\sum_{s=1}^{\hat{j}}\eta_{s}=1$. The expert evaluation matrix is described as

$$\left[\begin{array}{cccc} \left(E_{11}\left(q\right),F_{11}\left(q\right),G_{11}\left(q\right)\right) & \left(E_{12}\left(q\right),F_{12}\left(q\right),G_{12}\left(q\right)\right) & \cdots & \left(E_{1h}\left(q\right),F_{1h}\left(q\right),G_{1h}\left(q\right)\right)\\ \left(E_{21}\left(q\right),F_{21}\left(q\right),G_{21}\left(q\right)\right) & \left(E_{22}\left(q\right),F_{22}\left(q\right),G_{22}\left(q\right)\right) & \cdots & \left(E_{2h}\left(q\right),F_{2h}\left(q\right),G_{2h}\left(q\right)\right)\\ \left(E_{31}\left(q\right),F_{31}\left(q\right),G_{31}\left(q\right)\right) & \left(E_{32}\left(q\right),F_{32}\left(q\right),G_{32}\left(q\right)\right) & \cdots & \left(E_{3h}\left(q\right),F_{3h}\left(q\right),G_{3h}\left(q\right)\right)\\ \vdots & \vdots & \ddots & \vdots \\ \left(E_{g1}\left(q\right),F_{g1}\left(q\right),G_{g1}\left(q\right)\right) & \left(E_{g2}\left(q\right),F_{g2}\left(q\right),G_{g2}\left(q\right)\right) & \cdots & \left(E_{gh}\left(q\right),F_{gh}\left(q\right),G_{gh}\left(q\right)\right) \end{array}\right]$$

where $\left(E_{gh}\left(q\right),F_{gh}\left(q\right),G_{gh}\left(q\right)\right)$ are the three sets of some values in [0,1], denoted the positive, neutral, and negative membership grades with the constraint $0\leq {\left(u^{+}\right)}^{2}+{\left(v^{+}\right)}^{2}+{\left(w^{+}\right)}^{2}\leq 1,$ for all q∈N, such that

$$u^{+}=\underset{u\in E_{C}\left(g\right)}{⋃}\max\left\{u\right\},\;\delta^{+}=\underset{v\in F_{C}\left(g\right)}{⋃}\max\left\{v\right\},\;\mathrm{and}\;\partial^{+}=\underset{\;\;w\in G_{C}\left(g\right)}{⋃}\max\left\{w\right\}.$$

**Step-1**  Construct the expert evaluation matrix ${\left(R\right)}^{{}_{\hat{j}}}$.
  $$\left[\begin{array}{cccc} \left(E_{11}^{{}_{\hat{j}}}\left(q\right),F_{11}^{{}_{\hat{j}}}\left(q\right),G_{11}^{{}_{\hat{j}}}\left(q\right)\right) & \left(E_{12}^{{}_{\hat{j}}}\left(q\right),F_{12}^{{}_{\hat{j}}}\left(q\right),G_{12}^{{}_{\hat{j}}}\left(q\right)\right) & \cdots & \left(E_{1h}^{{}_{\hat{j}}}\left(q\right),F_{1h}^{{}_{\hat{j}}}\left(q\right),G_{1h}^{{}_{\hat{j}}}\left(q\right)\right)\\ \left(E_{21}^{{}_{\hat{j}}}\left(q\right),F_{21}^{{}_{\hat{j}}}\left(q\right),G_{21}^{{}_{\hat{j}}}\left(q\right)\right) & \left(E_{22}^{{}_{\hat{j}}}\left(q\right),F_{22}^{{}_{\hat{j}}}\left(q\right),G_{22}^{{}_{\hat{j}}}\left(q\right)\right) & \cdots & \left(E_{2h}^{{}_{\hat{j}}}\left(q\right),F_{2h}^{{}_{\hat{j}}}\left(q\right),G_{2h}^{{}_{\hat{j}}}\left(q\right)\right)\\ \left(E_{31}^{{}_{\hat{j}}}\left(q\right),F_{31}^{{}_{\hat{j}}}\left(q\right),G_{31}^{{}_{\hat{j}}}\left(q\right)\right) & \left(E_{32}^{{}_{\hat{j}}}\left(q\right),F_{32}^{{}_{\hat{j}}}\left(q\right),G_{32}^{{}_{\hat{j}}}\left(q\right)\right) & \cdots & \left(E_{3h}^{{}_{\hat{j}}}\left(q\right),F_{3h}^{{}_{\hat{j}}}\left(q\right),G_{3h}^{{}_{\hat{j}}}\left(q\right)\right)\\ \vdots & \vdots & \ddots & \vdots \\ \left(E_{g1}^{{}_{\hat{j}}}\left(q\right),F_{g1}^{{}_{\hat{j}}}\left(q\right),G_{g1}^{{}_{\hat{j}}}\left(q\right)\right) & \left(E_{g2}^{{}_{\hat{j}}}\left(q\right),F_{g2}^{{}_{\hat{j}}}\left(q\right),G_{g2}^{{}_{\hat{j}}}\left(q\right)\right) & \cdots & \left(E_{gh}^{{}_{\hat{j}}}\left(q\right),F_{gh}^{{}_{\hat{j}}}\left(q\right),G_{gh}^{{}_{\hat{j}}}\left(q\right)\right) \end{array}\right]$$
  where ${}_{\hat{j}}$ represents the number of expert.
**Step-2**  Construct the normalized decision matrix ${\left(L\right)}^{\hat{j}}.$ Where
  $${\left(L\right)}^{\hat{j}}=\left\{\begin{array}{ccc} \left(E_{gh}^{{}_{\hat{j}}}\left(q\right),F_{gh}^{{}_{\hat{j}}}\left(q\right),G_{gh}^{{}_{\hat{j}}}\left(q\right)\right) & if & \mathrm{Benefit}\;\mathrm{type}\;\mathrm{criteria}\\ \left(G_{gh}^{{}_{\hat{j}}}\left(q\right),F_{gh}^{{}_{\hat{j}}}\left(q\right),E_{gh}^{{}_{\hat{j}}}\left(q\right)\right) & if & \mathrm{Cos}t\;\mathrm{type}\;\mathrm{criteria} \end{array}\right.$$
**Step-3**  Aggregate the individual decision matrices based on the spherical hesitant fuzzy aggregation operators to construct the collective matrix as follows.
  $$\left[\begin{array}{cccc} \left(E_{11}\left(q\right),F_{11}\left(q\right),G_{11}\left(q\right)\right) & \left(E_{12}\left(q\right),F_{12}\left(q\right),G_{12}\left(q\right)\right) & \cdots & \left(E_{1h}\left(q\right),F_{1h}\left(q\right),G_{1h}\left(q\right)\right)\\ \left(E_{21}\left(q\right),F_{21}\left(q\right),G_{21}\left(q\right)\right) & \left(E_{22}\left(q\right),F_{22}\left(q\right),G_{22}\left(q\right)\right) & \cdots & \left(E_{2h}\left(q\right),F_{2h}\left(q\right),G_{2h}\left(q\right)\right)\\ \left(E_{31}\left(q\right),F_{31}\left(q\right),G_{31}\left(q\right)\right) & \left(E_{32}\left(q\right),F_{32}\left(q\right),G_{32}\left(q\right)\right) & \cdots & \left(E_{3h}\left(q\right),F_{3h}\left(q\right),G_{3h}\left(q\right)\right)\\ \vdots & \vdots & \ddots & \vdots \\ \left(E_{g1}\left(q\right),F_{g1}\left(q\right),G_{g1}\left(q\right)\right) & \left(E_{g2}\left(q\right),F_{g2}\left(q\right),G_{g2}\left(q\right)\right) & \cdots & \left(E_{gh}\left(q\right),F_{gh}\left(q\right),G_{gh}\left(q\right)\right) \end{array}\right]$$
**Step-4**  In this step, we find the weights of each of the attribute by using the spherical hesitant fuzzy entropy.
  $$\gamma_{q}=\frac{1+\frac{1}{n}\sum_{i=1}^{h}\left(E_{i}\ln\left(E_{i}\right)+F_{i}\ln\left(F_{i}\right)+G_{i}\ln\left(G_{i}\right)\right)}{\sum_{q=1}^{h}\left(1+\frac{1}{n}\sum_{i=1}^{h}\left(E_{i}\ln\left(E_{i}\right)+F_{i}\ln\left(F_{i}\right)+G_{i}\ln\left(G_{i}\right)\right)\right)}$$
**Step-5**  Exploit the established aggregation operators to achieve the SHFN $X_{q}\left(q=1,2,\ldots .,m\right)$ for the alternatives $A_{q}$, that is, the established operators to obtained the collective overall preference values of $X_{q}\left(q=1,2,\ldots .,m\right)$ for the alternatives $A_{q}$, where ${\left(\beta_{1},\beta_{2},\ldots \beta_{m}\right)}^{T}$ is the weight vector of the attributes.
**Step-6**  Compute the score (According to Definition 15) of all the overall values $X_{q}\left(q=1,2,\ldots .,m\right)$ for the alternatives $A_{q}$.
**Step-7**  Rank the alternatives $A_{q}\left(q=1,2,\ldots .,m\right)$ and select the best one having the greater value.

## 6. Illustrative Example

**The hotel recommendation approach and its case study:**

TripAdvisor.com is one of the leading travel communities in the world, covering restaurants in more than 190 countries, with about 200 million global tourist ratings and reviews. TripAdvisor.com offers reviews and views of travel related content, such as hotels, restaurants, and attractions, as an American tourism website. In general, few local travelers use TripAdvisor.com to find restaurants instead of regional sites for catering services (like Yelp). Ultimately, when suggesting hotels, TripAdvisor.com does not further differentiate between independent visitors and local customers. The suggested model aims to help visitors find satisfactory restaurants at their destinations on TripAdvisor.com. This case study, as with TripAdvisor.com, is also carried out without distinguishing these two categories of users, and “tourist” applies to subjective users of the TripAdvisor.com website. In addition, TripAdvisor.com enables tourists to score restaurants on four distinct aspects of a 5-star marking system: atmosphere, food, service, and cleanliness [58].

A realistic MCGDM example of a new hotel recommendation approach based on customer online feedback using spherical hesitant fuzzy numbers SHFNs will be presented to demonstrate the effectiveness and supremacy of the investigated approach. Multi-criteria decision-making (MCDM) methods, as studied by many scholars, are frequently used in field research [58,59]. Indeed, various groups of consumers concentrate on different hotel characteristics, such as price, service, comfort level, etc. [60]. The most important factors were divided by Sohrabi et al. [59] into ten dimensions. In addition, some other studies indicate their views on the factors that have their own factors for customers to compare hotels on each tourism website. While various groups of consumers concentrate on different hotel requirements, the reason lies in the fact that getting a good rest is the main objective of consumers booking a hotel. It is therefore irrational to disregard the importance of both of the two classes and to combine the data using the weighted averaging (WA) and geometric (WG) method. We employ the Log−SHFWA and Log−SHFWG operator, an extension of SHFN, to combine the information in this study to cover this defect.

Many tourism websites nowadays allow consumers to perform online surveys. For example, on TripAdvisor.com, consumers are permitted to assess the hotel on the basis of four criteria, respectively, assigned to the atmosphere, food, service, and cleanliness. Correspondingly, in relation to these four criteria, we will receive online feedback from consumers and perform the data through logrithmic-SHFSs. The main aim of this study is to create a systematic model for decision support to help independent tourists choose restaurants on TripAdvisor.com using social information. For example, with respect to four requirements, tourists on TripAdvisor.com will rate restaurants, including atmosphere, food, service, and cleanliness [60].

Consider tourists choose a restaurant based on four alternatives {$A_{1},A_{2},A_{3,}A_{4}$} which are consider for further evaluation to choose the best optimal hotel for stay a night or two or so on days. Tourists on TripAdvisor.com can rate restaurants with respect to four criteria, which are $B_{1}=$**atmosphere**, $B_{2}=$**food**, $B_{3}=$**service**, and $B_{4}=$ **cleanliness** with weight vector ${\left(0.1,0.2,0.5,0.2\right)}^{T}$. The professional experts assessed their assessment report for each alternative against their corresponding criteria in the form of spherical hesitant fuzzy values. Now, we use the developed approach of SHF logarithmic weighted average operator to get the best SHPP system by utilizing the above step wise decision algorithm.

**Solution using by the developed Algorithm:**

**Step-1**  The expert evaluation information in the form of the spherical hesitant fuzzy sets is enclosed in Table 1:
**Step-2**  Normalized logarithmic spherical hesitant fuzzy decision matrix calculated in Table 2:
**Step-3**  As, in this problem, we consider only one expert, so we do not need to find the overall preference of the experts.
**Step-4**  The expert weight are given in this case study are ${\left(0.1,0.2,0.5,0.2\right)}^{T}$.
**Step-5**  Now, we calculate the aggregated values of each alternative under criteria weight vector using proposed list of logarithmic spherical hesitant fuzzy aggregation operators as follows:
**Case-1: Using** Log−SHFWA **aggregation operator;**
  $$\begin{array}{ccc} & & Log-SHFWA\left(C_{1,}C_{2}\ldots C_{m}\right)\\ & = & \underset{\left(u_{q},v_{q},w_{q}\right)\in \left(\begin{array}{c} Log_{i}E_{q},Log_{i}\left(\sqrt{1-F_{q}^{2}}\right),\\ Log_{i}\left(\sqrt{1-G_{q}^{2}}\right) \end{array}\right)}{⋃}\;\;\left\{\begin{array}{c} \sqrt{1-\prod_{q=1}^{m}{\left(Log_{i}u_{q})\right)}^{2\beta_{q}}},\\ \prod_{q=1}^{m}{\left(Log_{i}\left(\sqrt{1-v_{q}^{2}}\right)\right)}^{\beta_{q}},\\ \prod_{q=1}^{m}{\left(Log_{i}\left(\sqrt{1-w_{q}^{2}}\right)\right)}^{\beta_{q}} \end{array}\right\} \end{array}$$
  The aggregated values of each alternative using Log−SHFWA aggregation operator is enclosed in Table 3:
**Case-2: Using** Log−SHFWG **aggregation operator;**
  $$\begin{array}{ccc} & & Log-SHFWG\left(C_{1,}C_{2}\ldots C_{m}\right)\\ & = & \underset{\left(u_{q},v_{q},w_{q}\right)\in \left(\begin{array}{c} 1-Log_{{}_{i}}E_{q},Log_{{}_{i}}\left(1-F_{q}\right),\\ Log_{{}_{i}}\left(1-G_{q}\right) \end{array}\right)}{⋃}\left\{\begin{array}{c} {\left(\prod_{q=1}^{m}\sqrt{1-{\left(Log_{{}_{i}}u_{q}\right)}^{2}}\right)}^{\beta },\\ \sqrt{1-{\left(1-\prod_{q=1}^{m}{\left(Log_{{}_{i}}\sqrt{1-v_{q}^{2}}\right)}^{2}\right)}^{\beta }},\\ \sqrt{1-{\left(1-\prod_{q=1}^{m}{\left(Log_{{}_{i}}\sqrt{1-w_{q}^{2}}\right)}^{2}\right)}^{\beta }} \end{array}\right\}. \end{array}$$
  The aggregated values of each alternative using Log−SHFWG aggregation operator is enclosed in Table 4:
**Step-6**  Now, Score values of each alternative of aggregated information are enclosed in Table 5:
**Step-7**  The rank of the alternatives $A_{q}\left(q=1,2,3,4\right)$ is enclosed in Table 6:

## 7. Comparison Study

In this section, we established the comparison of the propose logarithmic aggregation operators based decision-making methodology and the existing technique based on sine trigonometric-based spherical hesitant fuzzy aggregation operators. For this purpose, we take the spherical hesitant fuzzy information form Naeem et al. [57] in Table 7. The attribute weight vector is ${\left(0.2,0.4,0.1,0.3\right)}^{T}.$

**Step-1**  The expert evaluation information [57] in the form of spherical hesitant fuzzy sets is enclosed in Table 7:

**Step-2**  The normalized expert evaluation information in enclosed in Table 8:

**Step-3**  As, in this problem, we consider only one expert, we do not need to find the overall preference of the experts.
**Step-4**  The expert weight are given in this case study are ${\left(0.2,0.4,0.1,0.3\right)}^{T}$.
**Step-5**  Now, we calculate the aggregated values of each alternative under criteria weight vector using logarithmic spherical hesitant fuzzy weighted averaging aggregation operators as follows:
  $$\begin{array}{ccc} & & Log-SHFWA\left(C_{1,}C_{2}\ldots C_{m}\right)\\ & = & \underset{\left(u_{q},v_{q},w_{q}\right)\in \left(Log_{i}E_{q},Log_{i}\left(\sqrt{1-F_{q}^{2}}\right),Log_{i}\left(\sqrt{1-G_{q}^{2}}\right)\right)}{⋃}\;\;\left\{\begin{array}{c} \sqrt{1-\prod_{q=1}^{m}{\left(Log_{i}u_{q})\right)}^{2\beta_{q}}},\\ \prod_{q=1}^{m}{\left(Log_{i}\left(\sqrt{1-v_{q}^{2}}\right)\right)}^{\beta_{q}},\\ \prod_{q=1}^{m}{\left(Log_{i}\left(\sqrt{1-w_{q}^{2}}\right)\right)}^{\beta_{q}} \end{array}\right\} \end{array}$$
  The collective overall preference values of each alternative using Log−SHFWA aggregation operator is enclosed in Table 9:

**Step-6**  Ranking result is enclosed in Table 10:

### Discussion

Here, we conducted a comparison of the established logarithmic function-based aggregation operators with existing sine trigonometric spherical hesitant fuzzy aggregation operators presented in [57], showing the strength to handle uncertainty in real-life decision-making problems (DMPs). The impressive point of this method is that it covers the valuation spaces of PyHFSs, PFSs, and SFSs because of its generalized structure. From Table 10, results shows that the proposed decision-making technique is valid and reliable to tackle the uncertainty in decision making problems. Our proposed method is applicable and appropriate for input data of all types. The model suggested is effective for addressing uncertainties. With the consideration of hesitation, this approach covers the area of IFS, PyFS, PFS, SFSs, and SHFSs. We may use our method effectively in different circumstances, in present work we apply it for best hotel selection. The proposed decision-making method is clear and simple, and can be easily extended to various results.

## 8. Reliability and Validity Test

Generally, it is enormously challenging to identify the optimum probable alternative among the specified group decision matrices. Wang and Triantaphyllou [61] started the model to evaluate the applicability and legitimacy of decision making procedures. The test stages are below.

**Test Step-1**: The suitable and active MAGDM methodology is that we interchange the normalized component for the worse component of the alternative by validating the preeminent probable alternative without any alteration and also without adjusting the relative status of every decision criterion.

**Test Step-2**: Transitive property must be met through an operative and proper MAGDM method.

**Test Step-3**: When a MAGDM problem is converted into minor problems. To rank the alternative, we put on alike method on minor problems which is used in MAGDM problem, a combined alternative rank should be identical with actual rank of un-decomposed problem.

Changed the specified MAGDM problem into a minor one and put on the similar suggested decision-making procedure to discover the finest outcome. The proper and active MAGDM methodology is that, if we put on the similar procedure to a minor problem, the outcome will be the identical as the MAGDM problem.

### Validity Test for Proposed Methodology

In this segment, we check the appropriation and authentication of the our suggested approach by utilizing validity and reliability test [61] conferred above. The normalized spherical hesitant fuzzy material is enclosed in the Table 11 as follows:

**Test** **step-1** In this step, we exchange the normalized element for the worse element of the alternative by demonstrating the best possible alternative without any adjustment and also without modifying the comparative status of each decision criterion. The updated decision matrix is calculated in Table 12:
  Now, we calculate the aggregated value of the each alternative under attribute weight vector is ${\left(0.1,0.2,0.5,0.2\right)}^{T}$ using proposed list of logarithmic spherical hesitant fuzzy aggregation operators as follows:
  **Case-1: Using**Log−SHFWA**aggregation operator:** The aggregated values of each alternative using Log−SHFWA aggregation operator is enclosed in Table 13:
  **Case-2: Using**Log−SHFWG**aggregation operator:** The aggregated values of each alternative using Log−SHFWA aggregation operator is enclosed in Table 14:
  Now, the Score of the aggregated values of each alternative is enclosed in Table 15:
  Rank the alternatives $A_{q}\left(q=1,2,3,4\right)$ is enclosed in Table 16:
  After applying the Test step-1, we obtained the same best alternative $A_{4}$ as we obtained in our proposed numerical case study.
**Test** **Step-2** **&** **3** Now, we check the step-2 and -3 of the validity test to show that the proposed methodology is effective and appropriate. For this, first we transformed the consider MAGDM problem into three smaller sub-problems as $\left\{A_{2},A_{3},A_{4}\right\}$, $\left\{A_{3},A_{4},A_{1}\right\}$, and $\left\{A_{3},A_{1},A_{2}\right\}$. Now, we apply the our proposed decision-making methodology on the smaller transformed problems and obtained the following ranking of the alternatives: $A_{3}>$ $A_{2}>A_{4},$ $A_{2}>$ $A_{4}>A_{1}$, and $A_{3}>A_{4}>A_{1}$, respectively. While assigning a comprehensive ranking, we find that $A_{3}>A_{2}>$ $A_{4}>A_{1}$ is the same as the standard decision-making methodology results.

## 9. Conclusions

This study introduces a comprehensive model for supporting decisions that uses social information to help independent tourists find satisfactory hotels on TripAdvisor.com. In general, the developed scheme fully utilizes social data, such as online reviews and social relationships, and it considers interdependence among criteria by utilizing novel logarithmic spherical fuzzy aggregation operators, as do traditional decision support modeling techniques. In addition, the proposed study presented the list of novel operation laws using logarithmic function to develop the list of logarithmic spherical hesitant fuzzy aggregation operators to tackle the uncertainty in real life decision making problems. A generalized decision-making algorithm is developed to address the multi-attribute decision-making problems. This study considers a case study of hotel selection based on the proposed logarithmic spherical hesitant fuzzy aggregation operators. The suggested hotel recommendation technique turns out to be more acceptable and reliable from the comparison study than the comparative methods. It also shows that our approach to hotel reviews is successful in addressing consumers’ customized demands. We concluded that customers pay more attention to two criteria—atmosphere and service—through the study of online reviews. Because of this, if it focuses on fixing the current problems in these two areas, the hotel will significantly increase hotel satisfaction.

In a future study, we will develop fuzzy decision-making techniques such as TOPSIS, TODAM, VIKOR, GRY, and EDAS methodologies to evaluate the appropriate hotel in any venue based on customer demand.

## Figures and Tables

**Table 1:** Expert Evaluation Information.

	$B_{1}$	$B_{2}$	$B_{3}$	$B_{4}$
$A_{1}$	$\left\{\left(0.25,0.32,0.35\right)\right\}$	$\left\{\left(0.27,0.26,0.42\right)\right\}$	$\left\{\left(0.38,0.25,0.61\right)\right\}$	$\left\{\left(0.26,0.25,0.41\right)\right\}$
$A_{2}$	$\left\{\left(0.34,0.15,0.17\right)\right\}$	$\left\{\left(0.43,0.33,0.52\right)\right\}$	$\left\{\left(0.36,0.21,0.41\right)\right\}$	$\left\{\left(0.42,0.14,0.61\right)\right\}$
$A_{3}$	$\left\{\left(0.23,0.11,0.54\right)\right\}$	$\left\{\left(0.26,0.21,0.31\right)\right\}$	$\left\{\left(0.33,0.23,0.43\right)\right\}$	$\left\{\left(0.35,0.32,0.44\right)\right\}$
$A_{4}$	$\left\{\left(0.37,0.25,0.62\right)\right\}$	$\left\{\left(0.43,0.22,0.51\right)\right\}$	$\left\{\left(0.43,0.34,0.52\right)\right\}$	$\left\{\left(0.43,0.24,0.62\right)\right\}$

**Table 2:** Normalized Expert Evaluation Information.

	$B_{1}$	$B_{2}$	$B_{3}$	$B_{4}$
$A_{1}$	$\left\{\left(0.35,0.32,0.25\right)\right\}$	$\left\{\left(0.42,0.26,0.27\right)\right\}$	$\left\{\left(0.61,0.25,0.38\right)\right\}$	$\left\{\left(0.41,0.25,0.26\right)\right\}$
$A_{2}$	$\left\{\left(0.34,0.15,0.17\right)\right\}$	$\left\{\left(0.52,0.33,0.43\right)\right\}$	$\left\{\left(0.41,0.21,0.36\right)\right\}$	$\left\{\left(0.61,0.14,0.42\right)\right\}$
$A_{3}$	$\left\{\left(0.54,0.11,0.23\right)\right\}$	$\left\{\left(0.31,0.21,0.26\right)\right\}$	$\left\{\left(0.43,0.23,0.33\right)\right\}$	$\left\{\left(0.44,0.32,0.35\right)\right\}$
$A_{4}$	$\left\{\left(0.62,0.25,0.37\right)\right\}$	$\left\{\left(0.51,0.22,0.43\right)\right\}$	$\left\{\left(0.52,0.34,0.43\right)\right\}$	$\left\{\left(0.62,0.24,0.43\right)\right\}$

**Table 3:** Aggregated Values (Log−SHFWG).

$A_{1}$	0.9089,0.2071,0.2557
$A_{2}$	0.8818,0.1636,0.2920
$A_{3}$	0.8420,0.1792,0.2483
$A_{4}$	0.9282,0.2272,0.3506

**Table 4:** Aggregated Values (Log−SHFWG).

$A_{1}$	0.4320,0.1858,0.9667
$A_{2}$	0.3839,0.1434,0.9563
$A_{3}$	0.3340,0.1586,0.9687
$A_{4}$	0.4926,0.2066,0.9365

**Table 5:** Score Values.

Operators	$\mathrm{Sc}\left(A_{1}\right)$	$\mathrm{Sc}\left(A_{2}\right)$	$\mathrm{Sc}\left(A_{3}\right)$	$\mathrm{Sc}\left(A_{4}\right)$
Log−SHFWA	0.9958	0.9930	0.9880	0.9959
Log−SHFWG	0.0107	0.0623.	−0.2138	0.3823

**Table 6:** Ranking of the alternatives.

Operators	Score	Best Alternative
Log−SHFWA	$Sc\left(A_{4}\right)>Sc\left(A_{1}\right)>Sc\left(A_{2}\right)>Sc\left(A_{3}\right)$	$A_{4}$
Log−SHFWG	$Sc\left(A_{4}\right)>Sc\left(A_{2}\right)>Sc\left(A_{1}\right)>Sc\left(A_{3}\right)$	$A_{4}$

**Table 7:** Expert Evaluation Information.

	$V_{1}$	$V_{2}$	$V_{3}$	$V_{4}$
$G_{1}$	$\left\{\left(0.3,0.2,0.4\right)\right\}$	$\left\{\left(0.2,0.6,0.5\right)\right\}$	$\left\{\left(0.1,0.5,0.3\right)\right\}$	$\left\{\begin{array}{c} \left(0.1,0.5,0.6\right),\\ \left(0.3,0.4,0.5\right) \end{array}\right\}$
$G_{2}$	$\left\{\left(0.1,0.5,0.2\right)\right\}$	$\left\{\left(0.2,0.3,0.4\right)\right\}$	$\left\{\begin{array}{c} \left(0.1,0.1,0.6\right),\\ \left(0.3,0.1,0.4\right) \end{array}\right\}$	$\left\{\left(0.1,0.4,0.2\right)\right\}$
$G_{3}$	$\left\{\left(0.4,0.1,0.5\right)\right\}$	$\left\{\begin{array}{c} \left(0.1,0.1,0.6\right),\\ \left(0.3,0.2,0.4\right) \end{array}\right\}$	$\left\{\left(0.4,0.2,0.5\right)\right\}$	$\left\{\left(0.4,0.2,0.5\right)\right\}$
$G_{4}$	$\left\{\left(0.2,0.2,0.3\right)\right\}$	$\left\{\left(0.1,0.2,0.3\right)\right\}$	$\left\{\left(0.2,0.4,0.3\right),\left(0.4,0.4,0.6\right)\right\}$	$\left\{\left(0.2,0.4,0.3\right)\right\}$

**Table 8:** Normalized Expert Evaluation Information.

	$V_{1}$	$V_{2}$	$V_{3}$	$V_{4}$
$G_{1}$	$\left\{\left(0.4,0.2,0.3\right)\right\}$	$\left\{\left(0.5,0.6,0.2\right)\right\}$	$\left\{\left(0.3,0.5,0.1\right)\right\}$	$\left\{\begin{array}{c} \left(0.6,0.5,0.1\right),\\ \left(0.5,0.4,0.3\right) \end{array}\right\}$
$G_{2}$	$\left\{\left(0.2,0.5,0.1\right)\right\}$	$\left\{\left(0.4,0.3,0.2\right)\right\}$	$\left\{\begin{array}{c} \left(0.6,0.1,0.1\right),\\ \left(0.4,0.1,0.3\right) \end{array}\right\}$	$\left\{\left(0.2,0.4,0.1\right)\right\}$
$G_{3}$	$\left\{\left(0.5,0.1,0.4\right)\right\}$	$\left\{\begin{array}{c} \left(0.6,0.1,0.1\right),\\ \left(0.4,0.2,0.3\right) \end{array}\right\}$	$\left\{\left(0.5,0.2,0.4\right)\right\}$	$\left\{\left(0.5,0.2,0.4\right)\right\}$
$G_{4}$	$\left\{\left(0.3,0.2,0.2\right)\right\}$	$\left\{\left(0.3,0.2,0.1\right)\right\}$	$\left\{\left(0.3,0.4,0.2\right),\left(0.6,0.4,0.4\right)\right\}$	$\left\{\left(0.3,0.4,0.2\right)\right\}$

**Table 9:** Overall Preference Value (Log−SHFWA).

$G_{1}$	$\left\{0.8984,0.3797,0.1308\right\},\left\{0.8766,0.3523,0.1830\right\}$
$G_{2}$	$\left\{0.7026,0.2654,0.1046\right\},\left\{0.6564,0.2654,0.1170\right\}$
$G_{3}$	$\left\{0.9245,0.1046,0.1860\right\},\left\{0.8765,0.1384,0.2912\right\}$
$G_{4}$	$\left\{0.6637,0.2129,0.1203\right\},\left\{0.7270,0.2129,0.1294\right\}$

**Table 10:** Score Values.

Operators	$\mathrm{Sc}\left(G_{1}\right)$	$\mathrm{Sc}\left(G_{2}\right)$	$\mathrm{Sc}\left(G_{3}\right)$	$\mathrm{Sc}\left(G_{4}\right)$	Ranking of the Alternatives
ST−SHFWA [57]	0.4505	0.2463	0.4983	0.2332	$Sc\left(A_{3}\right)>Sc\left(A_{1}\right)>Sc\left(A_{2}\right)>Sc\left(A_{4}\right)$
Log−SHFWA	0.3646	0.3033	0.5404	0.3576	$Sc\left(A_{3}\right)>Sc\left(A_{1}\right)>Sc\left(A_{4}\right)>Sc\left(A_{2}\right)$

**Table 11:** Normalized Expert Evaluation Information (as Table 2).

	$B_{1}$	$B_{2}$	$B_{3}$	$B_{4}$
$A_{1}$	$\left\{\left(0.35,0.32,0.25\right)\right\}$	$\left\{\left(0.42,0.26,0.27\right)\right\}$	$\left\{\left(0.61,0.25,0.38\right)\right\}$	$\left\{\left(0.41,0.25,0.26\right)\right\}$
$A_{2}$	$\left\{\left(0.34,0.15,0.17\right)\right\}$	$\left\{\left(0.52,0.33,0.43\right)\right\}$	$\left\{\left(0.41,0.21,0.36\right)\right\}$	$\left\{\left(0.61,0.14,0.42\right)\right\}$
$A_{3}$	$\left\{\left(0.54,0.11,0.23\right)\right\}$	$\left\{\left(0.31,0.21,0.26\right)\right\}$	$\left\{\left(0.43,0.23,0.33\right)\right\}$	$\left\{\left(0.44,0.32,0.35\right)\right\}$
$A_{4}$	$\left\{\left(0.62,0.25,0.37\right)\right\}$	$\left\{\left(0.51,0.22,0.43\right)\right\}$	$\left\{\left(0.52,0.34,0.43\right)\right\}$	$\left\{\left(0.62,0.24,0.43\right)\right\}$

**Table 12:** Updated Normalized Expert Evaluation Information.

	$B_{1}$	$B_{2}$	$B_{3}$	$B_{4}$
$A_{1}$	$\left\{\left(0.35,0.32,0.25\right)\right\}$	$\left\{\left(0.27,0.26,0.42\right)\right\}$	$\left\{\left(0.61,0.25,0.38\right)\right\}$	$\left\{\left(0.26,0.25,0.41\right)\right\}$
$A_{2}$	$\left\{\left(0.34,0.15,0.17\right)\right\}$	$\left\{\left(0.43,0.33,0.52\right)\right\}$	$\left\{\left(0.41,0.21,0.36\right)\right\}$	$\left\{\left(0.42,0.14,0.61\right)\right\}$
$A_{3}$	$\left\{\left(0.23,0.11,0.54\right)\right\}$	$\left\{\left(0.31,0.21,0.26\right)\right\}$	$\left\{\left(0.33,0.23,0.43\right)\right\}$	$\left\{\left(0.44,0.32,0.35\right)\right\}$
$A_{4}$	$\left\{\left(0.37,0.25,0.62\right)\right\}$	$\left\{\left(0.51,0.22,0.43\right)\right\}$	$\left\{\left(0.43,0.34,0.52\right)\right\}$	$\left\{\left(0.62,0.24,0.43\right)\right\}$

**Table 13:** Aggregated Value (Log−SHFWA).

$A_{1}$	$\left\{0.8707,0.2071,0.3095\right\}$
$A_{2}$	$\left\{0.8316,0.1636,0.3328\right\}$
$A_{3}$	$\left\{0.7382,0.1792,0.3143\right\}$
$A_{4}$	$\left\{0.8906,0.2272,0.4147\right\}$

**Table 14:** Aggregated Value (Log−SHFWG).

$A_{1}$	$\left\{0.3459,0.1858,0.9509\right\}$
$A_{2}$	$\left\{0.3262,0.1434,0.9430\right\}$
$A_{3}$	$\left\{0.2529,0.1586,0.9493\right\}$
$A_{4}$	$\left\{0.4003,0.2066,0.9099\right\}$

**Table 15:** Score.

Operators	$\mathrm{Sc}\left(A_{1}\right)$	$\mathrm{Sc}\left(A_{2}\right)$	$\mathrm{Sc}\left(A_{3}\right)$	$\mathrm{Sc}\left(A_{4}\right)$
Log−SHFWA	0.9914	0.9854	0.9632	0.9911
Log−SHFWG	0.0337	0.0481	−0.2466	0.377

**Table 16:** Ranking of the alternatives.

Operators	Score	Best Alternative
Log−SHFWA	$Sc\left(A_{1}\right)>Sc\left(A_{4}\right)>Sc\left(A_{2}\right)>Sc\left(A_{3}\right)$	
Log−SHFWG	$Sc\left(A_{4}\right)>Sc\left(A_{2}\right)>Sc\left(A_{1}\right)>Sc\left(A_{3}\right)$	

## Data Availability

There is no associate Data.
